# Assessing Near‐Source Health and Equity Impacts of Liquefied Natural Gas Terminals

**DOI:** 10.1029/2025GH001609

**Published:** 2026-04-13

**Authors:** Xinran Wu, Tracey Holloway, Drashti Amin, Paul Meier, Vijay S. Limaye, Ade Samuel

**Affiliations:** ^1^ Nelson Institute for Environmental Studies, Center for Sustainability and the Global Environment University of Wisconsin‐Madison Madison WI USA; ^2^ Department of Atmospheric and Oceanic Sciences University of Wisconsin‐Madison Madison WI USA; ^3^ Natural Resources Defense Council New York NY USA; ^4^ Department of Population Health Sciences University of Wisconsin‐Madison Madison WI USA

**Keywords:** Clean Air Act, atmospheric dispersion, public health, environmental justice, industrial emissions

## Abstract

Growing global demand for natural gas has driven the expansion of liquefied natural gas (LNG) export terminals, which emit pollutants that can pose health risks to nearby communities. This study presents a novel modeling framework using the AMS/EPA Regulatory Model (AERMOD) to assess near‐source nitrogen dioxide (NO_2_) exposure, health impacts, and equity implications at the block‐group level. We apply this methodology to four LNG export terminals in the United States, simulating NO_2_ concentrations within a 50 km radius. Results show that LNG terminals substantially contribute to near‐source air pollution, with simulated 1‐hr maximum NO_2_ concentrations reaching up to 16% of the EPA's National Ambient Air Quality Standard (100 ppb). Site‐specific maximum concentrations were 15.7 ppb (Site A), 1.6 ppb (B), 10.7 ppb (C), and 0.3 ppb (D). Comparing NO_2_ concentrations with demographic patterns, Sites A and D showed higher concentrations, higher proportions of People of Color and low‐income populations, and greater health burdens in communities closer to the LNG facilities, indicating potential disproportionate impacts. The other sites showed weak or no spatial inequity patterns. Estimated annual NO_2_‐attributable all‐cause mortality rates per 100,000 people were 8.2 (A), 0.6 (B), 2.2 (C), and 0.1 (D); annual NO_2_‐attributable pediatric asthma rates per 100,000 children were 75.5 (A), 6.2 (B), 21.8 (C), and 1.1 (D). This study demonstrates how regulatory dispersion models like AERMOD can be adapted to evaluate near‐source health and equity impacts of industrial emissions and offers a transferable methodology for similar analyses across other high‐emitting facilities.

## Plain Language Summary

Liquefied natural gas (LNG) export terminals emit nitrogen dioxide (NO_2_), a pollutant linked to health risks. This study developed a methodology to evaluate near‐source health and equity impacts with the regulatory dispersion model, AMS/EPA Regulatory Model, and applied it to four U.S. LNG export terminals. We modeled NO_2_ levels near the terminals. We further conducted equity analysis comparing NO_2_ exposure with the proportion of people of color and low‐income populations at the block group level. We finally estimated potential health impacts from facility emissions, measured as NO_2_‐attributable all‐cause mortality and pediatric asthma.

## Key Points

- Near‐source health and equity impacts can be assessed with the regulatory dispersion model, AMS/EPA Regulatory Model, applied to four liquefied natural gas export terminals
- Modeled maximum 1‐hr NO_2_ concentrations range from 0.3 to 15.7 ppb across sites, reaching up to 16% of EPA 1‐hr standard of 100 ppb
- Estimated NO_2_‐attributable all‐cause mortality ranges from 0.1 to 8.2 deaths annually per 100,000 people living within a radius of 50 km

## 1 Introduction

Growing demand for natural gas has led to an expansion of the liquefied natural gas (LNG) industry (Zou et al., 2022). Liquefied natural gas is a form of natural gas that is cooled and condensed as a liquid, decreasing its volume by a factor of 600 for more cost‐effective transport (Abdul‐Wahab et al., 2020). LNG export terminals process natural gas into LNG for transport onto marine vessels (Ruszel, 2022). Creating LNG requires an energy‐intensive liquefaction process, consuming 200 to 400 kilowatt‐hours (kWh) of energy per ton of LNG produced, usually powered by natural gas combustion (Pospíšil et al., 2019; U.S. Department of Energy, 2014). Like other natural gas combustion sources, the energy generation and operations of LNG terminals emits nitrogen oxides (NO_*x*_, defined as the sum of nitrogen oxide, NO, and nitrogen dioxide, NO_2_), carbon monoxide (CO), fine particulate matter (PM_2.5_), volatile organic compounds, and other chemical species, including methane and ammonia (NH_3_) (Buonocore et al., 2023).

Among these pollutants, this study assesses LNG contributions to NO_2_ exposure and health impacts. According to the most recent United States Environmental Protection Agency (U.S. EPA) National Emissions Inventory, NO_*x*_ emissions from the four LNG terminals modeled in this study are approximately 220 times greater than sulfur dioxide (SO_2_) emissions and about 15 times greater than primary PM_2.5_ emissions (Abdul‐Wahab et al., 2020; Al‐Yafei et al., 2021; Gutiérrez et al., 2025). Direct exposure to NO_2_, especially in communities close to emission sources, has been linked to respiratory morbidity, including impaired host defense systems, increased lung inflammation, and decreased lung function and growth (Boningari & Smirniotis, 2016; Peel et al., 2013). As a precursor to ground‐level ozone (Mauzerall et al., 2005), NO_2_ also contributes to increased circulatory and respiratory mortality, as well as neurological disorders such as Alzheimer's and Parkinson's disease associated with ozone exposure (Jung et al., 2015; Kirrane et al., 2015; Turner et al., 2016). NO_2_ also contributes to the formation of secondary inorganic aerosols (nitrate), which is a major component of PM_2.5_ (T. Liu et al., 2021; Zhang et al., 2022). Additionally, a recent assessment by the U.S. Department of Energy (DOE) indicated that research is limited on the health effects of harmful air emissions (including NO_*x*_) emitted by LNG facilities (U.S. Department of Energy, 2024).

Beyond the direct impacts of LNG on air quality and health, the rapid expansion of LNG facilities has raised concerns about environmental justice and inequitable harms. A recent report from the U.S. DOE emphasized the lack of published literature that focuses on the impacts of LNG export operations including natural gas flaring and other associated emissions on local communities (U.S. Department of Energy, 2024). The concept of environmental justice emerged from the recognition that pollution disproportionately affects low‐income and communities of color across the U.S. (Banzhaf et al., 2019; Bullard, 2018; Galvin, 2020; Mohai et al., 2009). Studies have demonstrated that every stage of the fossil fuel life cycle (Epstein et al., 2011; O’Rourke & Connolly, 2003), including extraction (Johnston et al., 2019), processing and transportation (Ragothaman & Anderson, 2017; Williams et al., 2020; Yuan et al., 2018), and combustion (Cushing et al., 2023; J. Liu et al., 2021; Mikati et al., 2018; C. W. Tessum et al., 2021), contributes to these inequities, exposing disadvantaged populations to elevated levels of air pollution. This pattern has been described as fossil fuel racism (Donaghy et al., 2023). Many LNG export terminals are sited in communities already overburdened by industrial pollution from coal‐fired power plants, oil refineries, and other heavy industries (Donaghy et al., 2023; Johnson et al., 2014; Terrell & Julien, 2023; Terrell & St Julien, 2022). While an emerging body of literature suggests that fossil fuel phase‐outs could help alleviate these environmental injustices (Baker et al., 2021; Bazilian et al., 2021; Carley & Konisky, 2020), the expansion of LNG infrastructure raises concerns about perpetuating patterns of environmental inequity (Kemfert et al., 2022).

Equity and health impacts have been assessed using air quality models that simulate emissions into ground‐level ambient air concentrations, and overlay with demographic data and health metrics for equity and health implications (Bell & Ebisu, 2012; Hurbain et al., 2024; Southerland et al., 2021). However, modeling pollution exposure for equity impacts in fine demographic scales remains challenging (Clark et al., 2022; Gohlke et al., 2023; Paolella et al., 2018). Many air quality models lack the spatial resolution necessary to capture near‐source concentration hotspots that disproportionately affect vulnerable communities, such as the Community Multiscale Air Quality (CMAQ) model, which is computationally expensive and runs simulations with resolutions of 4–36 km (U.S. EPA, 2025c). While models like the Intervention Model for Air Pollution (InMAP) are specifically designed to support environmental justice analysis (Tessum et al., 2016, 2017), they do not allow for time‐varying analysis (only considering annual mean exposures), and do not evaluate gas‐phase pollutants (rather, they are restricted to PM_2.5_ and its component species) (Tessum et al., 2017). The relevance of InMAP to localized sources is also subject to potentially large biases (Jackson et al., 2023; R. Wu et al., 2021). Models like CMAQ and the Research LINE source (RLINE) and gridded data from satellite observations can also be used for environmental justice studies (Kerr et al., 2021; Valencia et al., 2023), but achieving fine‐scale, neighborhood‐level resolution often requires hybrid approaches or additional processing to integrate different data sources.

Studies have assessed the air quality, health, and equity implications of LNG infrastructure with varying methods. Al‐Yafei et al. applied a Life Cycle Assessment (LCA) approach to LNG supply chains in Qatar and found that LNG export terminals were among the largest contributors to air emissions and health impacts across all LNG supply chain stages (Al‐Yafei et al., 2021). However, the LCA method is based on aggregated emission factors and do not reveal spatial variation needed for characterizing local exposure. Abdul‐Wahab et al. used the CALPUFF dispersion model to simulate NO_2_ and CO concentrations of the Oman LNG facility. Their results showed that modeled NO_2_ concentrations exceeded U.S. EPA National Ambient Air Quality Standards (NAAQS) near the site (Abdul‐Wahab et al., 2020). Saha et al. used the EPA's Environmental Justice Screening and Mapping Tool (EJScreen) to assess the cumulative environmental and demographic burdens near LNG export terminals in Texas and Louisiana. By comparing EJScreen indicators for pollution, social vulnerability, and health risks, they found that nearby communities had disproportionately high burdens relative to state and national benchmarks (Saha et al., 2024). However, the EJScreen model assesses emissions only, lacking spatially‐resolved dispersion details. A study by Greenpeace and the Sierra Club employed the EPA Co‐benefits Risk Assessment (COBRA) screening model (U.S. EPA, 2025d), to quantify the health and economic impacts of permitted emissions from U.S. LNG export terminals (Heureaux‐Torres et al., 2024). This study estimated approximately 60 premature deaths and $957 million in annual economic costs due to secondary PM_2.5_. However, COBRA models only annual average PM_2.5_ concentrations and is limited to county‐scale exposure estimates. While these studies have established the foundation of assessing LNG impacts, this study represents a methodological advance by applying a Gaussian dispersion model to capture spatially‐resolved, time‐varying, and pollutant‐specific concentrations, enabling block group–level exposure and disparities evaluation.

We develop and apply an analysis method for NO_2_ dispersion, which could be used for any primary pollutant exposure and health analysis at the block group level. We use the AMS/EPA Regulatory Model (AERMOD), which is already widely used in the U.S. and worldwide to model near‐source pollution impacts of large facilities, often as part of the regulatory air‐permitting process (Cimorelli et al., 2005). We apply AERMOD to generate plume dispersion‐based concentrations and assess the block‐group level health impacts of the LNG facility alone (contributing emissions from other nearby sources are not included) (Perry et al., 2005). In addition to its role in regulatory permitting, AERMOD has been widely used in research applications to quantify emission impacts from different industries (Adeniran et al., 2019; Amoatey et al., 2020; Craig et al., 2020; Radford et al., 2021); evaluate the effectiveness of emission reductions (Josimović et al., 2024); predict short‐term air quality (Kumar et al., 2017); and simulate concentration outputs for comparison with ground observations (Pandey et al., 2023; Salva et al., 2021).

Existing literature has reviewed or applied AERMOD for health risk or equity assessment. Tsai et al. used AERMOD to simulate concentrations of six hazardous species and calculated associated cancer risks in a heavy industry area in Taiwan (Tsai et al., 2019). Doost et al. applied AERMOD to model SO_2_ concentrations and associated health risks in a gas refinery in the Middle East over a 30 km radius and a receptor distance of 0.5 km (Doost et al., 2024). Arani et al. used AERMOD to model NO_2_ and SO_2_ dispersions of the steel and rolling industry over a 30 km radius and assessed associated health risks (Arani et al., 2021). Gardner‐Frolick et al. provided a framework for selecting modeling methods based on equity relevance, while Shan et al. assessed source‐specific exposure models for EJ and risk assessment (Gardner‐Frolick et al., 2022; Shan et al., 2024). Both reviews have suggested dispersion models like AERMOD as useful tools for providing fine‐scale concentration estimates for equity and environmental justice assessment. Maroko applied AERMOD to estimate PM_2.5_ from local stationary sources in New York City and examined disparities using tax‐lot level socio‐economic indicators, finding evidence of environmental injustice (Maroko, 2012). Lewis et al. used AERMOD to evaluate whether exposures to ammonia and hydrogen sulfide (H_2_S) from animal feeding operations varied across demographic groups at the block‐group level (Lewis et al., 2023). Collett et al. integrated field measurements and AERMOD modeling to examine population exposures to air emissions and noise from unconventional oil and gas development in Colorado (Collett et al., 2025).

Our study builds on previous work by considering affected communities at the block‐group level, particularly focusing on direct NO_2_ exposure, and health and equity impacts from LNG export terminals. To apply AERMOD to evaluate LNG health and equity impacts, we simulate NO_2_ concentrations at four existing LNG terminal facilities using AERMOD, then overlay simulated NO_2_ concentrations with block‐group demographic data to calculate exposed populations within a 50 km radius (the recommended range for AERMOD). Health risks are calculated using the risk factors in epidemiology studies and baseline mortality data aligned with the EPA BenMAP model. We introduce our Data and Methodology (Section 2), followed by Results (Section 3), Discussion (Section 4), and Conclusion (Section 5).

## 2 Data and Methodology

### 2.1 Data

We include four LNG export terminals (Site A–D) in this study based on their capacity and annual exports, as they account for a substantial share of total U.S. LNG export volumes and are large emitters of pollutants associated with LNG export operations. Details of each site's annual emissions, block group demographic characteristics, and general locations are summarized in Table S1 and Figure S1 in Supporting Information S1, respectively.

Emissions for NO_*x*_ are from the EPA 2022 Emissions Modeling Platform (EMP) facility‐level data (U.S. EPA, 2025a). The 2022 EMP data reports annual total emissions at industrial facility level, in tons per year (tons/year). These annual emissions are allocated to grams per second (g/s), assuming a constant emissions rate. The constant emissions rate represents a conservative estimate, recognizing that source variability may be high (Murphy & Allen, 2005; Rosselot et al., 2023).

Meteorology inputs include hourly surface observation data, collected by the National Weather Service (NWS) (NOAA, 2020a), and upper air radiosonde (sounding) data (NOAA, 2020b). Land cover and elevation data is from the United States Geological Survey (USGS) National Land Cover Database (USGS, 2020b), and the USGS National Elevation Data (NED) (USGS, 2020a), respectively. The land cover data is used for generating surface characteristics, and the elevation data is used to determine the elevations and hill heights for receptors and sources.

For equity and health impact analyses, we applied the United States Census Bureau American Community Survey (ACS) block‐group level age‐stratified population data (U.S. Census Bureau, 2020), EJScreen packaged data for block group shapefiles (U.S. EPA, 2025f), and census‐tract level age‐stratified baseline mortality rate data (U.S. EPA, 2025e).

### 2.2 Methodology

**Figure 1:**
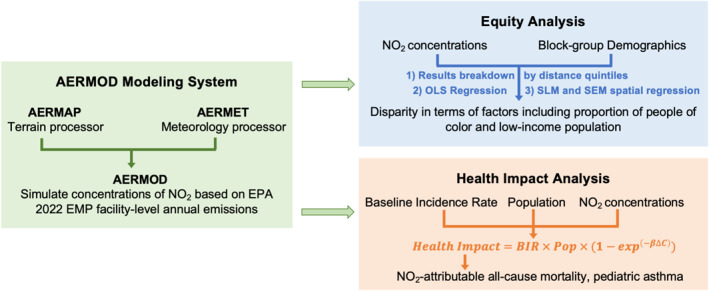
Methodology schematic overview.

The methodological workflow employed in this study is outlined in the schematic presented in Figure 1, comprises three modules: (a) AERMOD modeling where pollutant concentrations are simulated based on LNG‐related emissions; (b) health impact analysis where health risks are quantified based on baseline incidence rates and concentration–response functions (CRFs) from established epidemiology studies; and (c) equity analysis where modeled concentrations are linked with demographic data to assess associations between exposure and community characteristics, stratified by distance from the facility. We apply this methodology to the four LNG export terminals.

#### 2.2.1 AERMOD Modeling System

The AERMOD modeling system includes a dispersion model (AERMOD) and several data preprocessors to generate meteorological (AERMET) and terrain (AERMAP) conditions as inputs for the dispersion model. The AERMOD modeling framework applied in this study involves three steps: (a) process meteorology data with AERMET to generate key input files to AERMOD; (b) process elevation and land cover characteristics with AERMAP for use in AERMOD; and (c) compile and run AERMOD to get modeled concentrations for each pollutant. Each step's configurations, inputs, and outputs will be discussed below.

AERMET is the meteorological preprocessor which processes surface and upper‐air meteorological data to generate parameters for dispersion simulations in AERMOD, including surface temperature, wind speed and direction, friction velocity, Monin‐Obukhov length, convective velocity scale, and planetary boundary layer height, among others. Meteorological conditions, including wind, temperature, humidity, and cloud cover, are key factors that affect pollution dispersion in the atmosphere. We run AERMET (Version 23132) to process two types of data from the nearest weather stations of each studied site: (a) hourly surface observations collected by the NWS; (b) upper air radiosonde (sounding) data. Data processing takes two distinct stages in AERMET, the first stage extracts the surface and upper air data from meteorological files; the second stage calculates the boundary layer parameters based on the extracted outputs, and generates two AERMOD‐ready meteorological input files, one as the surface (.SFC) file, one as the profile (.PFL) file. For the surface file, parameters such as sensible heat flux, surface friction velocity, surface roughness length, albedo, and Bowen ratio are calculated and stored. The profile file includes parameters such as wind speed, wind direction, temperature, and standard deviations of the wind speed and direction fluctuations throughout the modeled year. Before running AERMET, the AERSURFACE (Version 20060) processor is applied to generate three surface parameters by analyzing land use and land cover data, including albedo, surface roughness length, and Bowen ratio, which directly affect the vertical profiles of temperature, wind, and turbulence, and then determine how pollutants disperse in the atmospheric boundary layer (U.S. EPA, 2022).

Meteorological data for 2018–2022 were processed with AERMET and merged into multi‐year surface and profile files. AERMOD was run using the 2022 emissions inventory applied across this 5‐year meteorological data set. The resulting annual average concentrations represent a multi‐year (2015–2022) average, consistent with EPA guidance recommending at least 5 years of meteorology to capture inter‐annual variability. These concentrations were used in all subsequent exposure, equity, and health impact analyses. Quality assurance for these inputs included standard AERMET checks, such as identifying missing or erroneous data and applying necessary adjustments or substitutions following EPA guidance.

AERMAP (Version 18081) is the terrain preprocessor which processes elevation data to be readily input into AERMOD. AERMAP produces terrain base elevations for each receptor and source and a hill height scale value for each receptor based on the NED data. Receptors can be specified in AERMAP as a polar grid or a Cartesian grid receptor network centered on the user‐specified source, or user‐specified discrete receptor locations using the Universal Transverse Mercator coordinates. In this study, we define the location of each LNG terminal site as source and the geometry centroids of all block groups within 50 km of each LNG site as receptors.

Finally, we run AERMOD (Version 23132) with the 2022 EPA EMP facility‐level annual emissions, processed meteorological and terrain inputs for each LNG site for NO_2_. We model the four LNG sites as point sources in our AERMOD simulations, as NO_*x*_ emissions from elevated stacks, such as combustion turbines, dominate total emissions. This assumption is further supported by the 2022 EMP data, which categorizes LNG NO_*x*_ emissions in the point source–oil and gas sector alongside other point sources.

While 2022 EMP facility‐level NO_*x*_ emissions include both NO and NO_2_, health‐relevant and regulatory assessments focus on ambient NO_2_ concentrations. For the modeling, we ran AERMOD with three different NO_*x*_‐to‐NO_2_ conversion schemes to simulate NO_2_ concentrations from NO_*x*_ emissions, explicitly accounting for the chemical conversion of NO to NO_2_ in the plume rather than assuming complete NO_*x*_‐to‐NO_2_ conversion. The three schemes evaluated are the Plume Volume Molar Ratio Method (PVMRM), Generic Reaction Set Method (GRSM), and Tiered Tracer Reaction Method 2 (TTRM2). We selected GRSM for the rest of health and equity analyses because it is the most recent EPA‐approved scheme and provides the most accurate representation of NO_*x*_ chemistry under conditions relevant to LNG terminal emissions (Stocker et al., 2023; U.S. EPA, 2024c). A detailed comparison and description of concentration ranges generated by the three schemes is presented in Figure S3 in Supporting Information S1. Ozone concentrations are incorporated in AERMOD under the PVMRM, GRSM, and TTRM2 schemes to account for plume entrainment and chemical interactions. For each LNG terminal site, we applied the annual arithmetic mean ozone concentrations of 36.8 ppb (Site A), 36.7 ppb (Site B), 33.6 ppb (Site C), and 37.8 ppb (Site D), based on observed data from the EPA Air Quality Monitoring network (U.S. EPA, 2025b). This approach ensures that the chemical transformation of NO_*x*_ in the plume reflects realistic ambient ozone conditions (U.S. EPA, 2004).

NO_2_ concentration outputs are annual highest 1‐hr daily maximum concentrations in μg/m^3^ at block group level, which are converted to parts per billion (ppb) based on 1 ppb = 1.88 μg/m^3^ at 1 atm and 25°C. The 1‐hr daily maximum NO_2_ concentrations are averaged annually to quantify health impacts. It is important to note that the Gaussian‐based structure of AERMOD simulates pollutant dispersion on an hourly basis, rather than capturing a dynamically evolving concentration field. The model does not account for background pollution or boundary inflow, so modeled results represent only the incremental impact of the LNG facility and do not reveal total air quality–related health burdens for affected communities.

#### 2.2.2 Health Impact Analysis

To assess the health impacts of pollution emitted from LNG export terminals, we followed similar methodologies as presented by Camilleri et al. and Anenberg et al., which estimated NO_2_‐attributable all‐cause mortality and pediatric asthma from all‐sourced NO_*x*_ emissions, respectively. We applied health risk functions and age‐stratified baseline mortality rates from epidemiological studies and data prepared by the Industrial Economics Inc. (IEc) based on the United States Small‐Area Life Expectancy Estimates Project (USALEEP) abridged life tables (National Center for Health Statistics, 2025). We estimated NO_2_‐attributable all‐cause mortality and NO_2_‐attributable pediatric asthma as two health impact endpoints (Anenberg et al., 2022; Chen et al., 2024). Other known health impacts of NO_2_ also include respiratory morbidity such as increased lung inflammation and decreased lung function and growth (Mauzerall et al., 2005).

The two NO_2_‐attributable health impacts for each block group within 50 km of each LNG terminal site are estimated using an epidemiologically‐derived log‐linear concentration‐response function (CRF) (Achakulwisut et al., 2019; Camilleri, Kerr, et al., 2023; Camilleri, Montgomery, et al., 2023; Health Effects Institute, 2022). We derive the concentration‐response coefficient, β, from a relative risk (RR) of 1.04 (95% confidence interval (CI): 1.02−1.06) per 10 μg/m^3^ from the 2022 Health Effects Institute (HEI) systematic review and meta‐analysis and previous studies for mortality and a RR of 1.26 (95% CI: 1.10−1.37) per 10 ppb for pediatric asthma (Anenberg et al., 2022), following Equation 1 (Health Effects Institute, 2022).

(1) $$\beta =\frac{\ln\left(RR\right)}{\Delta C}$$

where β is the concentration‐response coefficient; RR is the RR, ΔC is per 10 μg/m^3^ or ppb.

To account for variability in baseline incidence rates across block groups and demographic characteristics, we use census‐tract level age‐specific baseline all‐cause mortality rates (in deaths per 100,000 people per year) for each 10‐year age group, consistent with BenMAP‐CE's methodology. The baseline asthma incidence rates are state‐level data obtained from the Global Burden of Disease (GBD) Study 2021 done by the Institute for Health Metrics and Evaluation (GBD, 2021, 2025; Oh et al., 2025). These rates were applied to block‐group level population data stratified by age from the ACS to estimate block‐group level annual NO_2_‐attributable all‐cause mortality and pediatric asthma (for age group 0–14 years), following Equation 2.

(2) $${\text{NO}}_{2}-\text{Attributable}\;\text{Incidence}=\text{BIR}\times \text{Pop}\times \left(1-\text{ex}p^{\left(-\beta \Delta C\right)}\right)$$

where ${\text{NO}}_{2}-\text{Attributable}\;\text{Incidence}$ refers to the number of NO_2_‐attributable all‐cause mortality deaths and pediatric asthma cases; BIR is the age‐specific baseline incidence rate for each block groups; Pop is the exposed population of certain age group within each block group; β is the concentration‐response coefficient, which is 0.003922 per μg/m^3^ derived from a RR of 1.04 per 10 μg/m^3^ for mortality and 0.02311 per ppb derived from a RR of 1.26 per 10 ppb for pediatric asthma; ΔC is the annual‐averaged NO_2_ concentration change in μg/m^3^ or ppb at block group level.

#### 2.2.3 Equity Analysis

AERMOD concentration outputs at the block group level were combined with demographic data to conduct an equity analysis of exposed populations within a 50 km radius from the source. To characterize how exposure varies with proximity to the emission source, we categorized block groups into concentric distance quintiles (each representing 20% of block groups ordered by increasing distance). For each quintile, we computed summary statistics for modeled NO_2_ concentrations and key demographic indicators—including the proportion of People of Color (POC%), the proportion of low‐income population (LI%), and the proportion of elderly population, based on the EJScreen packaged data for block group shapefiles. The People of Color population is defined as all individuals who do not identify as non‐Hispanic White, and the low‐income population is defined as the population in households where the household income is less than or equal to twice the federal poverty level (U.S. EPA, 2024a). The elderly population is defined as people aged 65 or older, based on age breakdowns of the demographic data. We also compared estimated NO_2_‐attributable mortality and pediatric asthma rates across quintiles.

To further evaluate the statistical relationship between exposure and demographic characteristics, we applied a set of regression diagnostics and models. We first used the Ordinary Least Squares (OLS) model relating NO_2_ concentrations to demographic variables, then assessed spatial dependence in residuals using Moran's I. To consider potential spatial autocorrelation, we additionally fit both a Spatial Lag Model (SLM) and a Spatial Error Model (SEM) to obtain unbiased coefficient estimates, following the methodology of previous spatial regression studies (Mathieu et al., 2023; Scolio et al., 2024) Key regression coefficients and parameters for OLS, SLM, and SEM for each site are summarized in Table S3 in Supporting Information S1, followed by discussion on the interpretation.

## 3 Results

### 3.1 Modeled Concentrations

**Figure 2:**
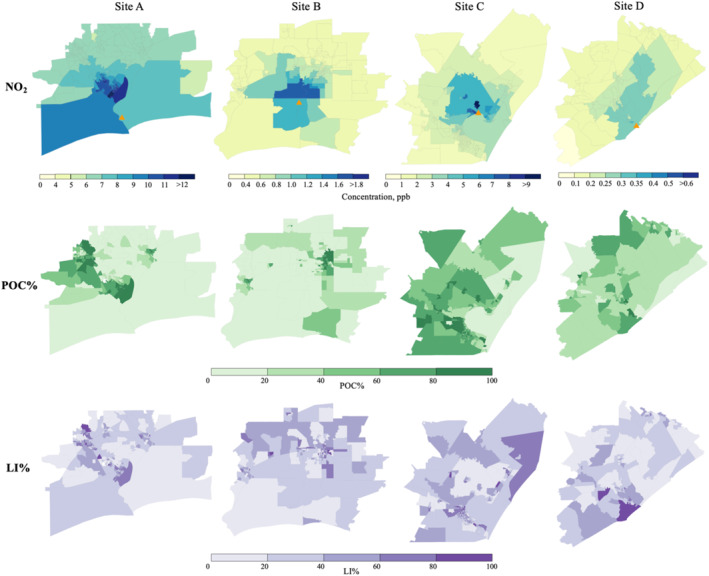
Modeled concentrations for NO_2_ (top), proportion of People of Color (POC%) (middle), and the proportion of low‐income population (LI%) (bottom) within each block group for sites A–D at block group level. Each column represents one liquefied natural gas (LNG) site. Concentration units are micrograms per cubic meter, μg/m^3^; POC% and LI% units are percentage (%). Orange triangles mark the locations of the LNG terminals.

Figure 2 (top panel) shows modeled NO_2_ concentrations at the block‐group level for each LNG site. Site A exhibits the largest range of daily 1‐hr maximum NO_2_ concentrations, from 5.8 to 15.7 ppb (averaging 7.7 ppb). Sites B and C show lower concentration ranges, with Site B's daily maximum NO_2_ ranging from 0.4 to 1.6 ppb (averaging 0.7 ppb) and Site C's ranging from 1.7 to 10.7 ppb (averaging 3.1 ppb). Site D exhibits the lowest modeled NO_2_ concentrations, ranging from 0.09 to 0.3 ppb (average of 0.2 ppb). Across the four sites, the ratio of maximum‐to‐minimum concentrations ranged from three (Sites A and D) to six (Site C), with Site B having a ratio of four.

The spatial concentration patterns demonstrate the capability of AERMOD to capture near‐source dispersion from LNG terminal emissions and reflect the influence of prevailing wind directions, as shown in the wind rose diagrams (Figure S3 in Supporting Information S1). Overall, within each modeling domain, NO_2_ concentrations decline with distance from the source, with the highest concentrations occurring near the LNG emission sites. At all sites, where southern, southeastern, and southwestern winds dominate, the highest NO_2_ concentrations are observed directly in downwind directions, clearly aligning with the prevailing wind patterns. For example, at Sites C and D, plumes extend predominantly northward from the source under prevailing southerly winds, while at Sites A and B, stronger southern and northeastern winds shape the observed impact area around the facility. However, it is important to note that, while wind roses indicate the prevailing frequency of wind directions, the modeled dispersion patterns are also affected by factors including stack height, plume rise, and atmospheric stability.

To evaluate the alignment between NO_*x*_ emissions and resulting NO_2_ concentrations, here we introduce the Concentration‐to‐Emission Ratio (CER), defined as the modeled NO_2_ concentration divided by the annual NO_*x*_ emissions, reported as ppb per ton. This metric is calculated with the goal of providing a heuristic to support broader consideration of LNG near‐source impacts, and to compare AERMOD modeling results across other LNG sites globally. We calculate both the average (CER_avg_) and maximum CER values (CER_max_) for each site. The CER_avg_ values range from 1.35 × 10^−3^ to 11.2 × 10^−3^ ppb per ton, showing overall consistency across sites despite large differences in total emissions. Site A, with the highest NO_*x*_ emissions (4,267 tons/yr), has a CER_avg_ at 1.81 × 10^−3^ ppb per ton; Site B (505 tons/yr) has 1.35 × 10^−3^; and Site C (2045 tons/yr) has 1.48 × 10^−3^. The CER_max_ values are also relatively consistent across Sites A‐C, specifically, Site A has a CER_max_ of 3.67 × 10^−3^ (range: 1.37–3.67 × 10^−3^); Site B, 3.11 × 10^−3^ (0.87–3.11 × 10^−3^); and Site C, 5.21 × 10^−3^ (0.82–5.27 × 10^−3^). This consistency in both average and maximum CER values suggests a stable conversion from emissions to modeled ground‐level NO_2_ concentrations across varied site contexts. However, Site D (17.9 ton/yr) has CER values about one magnitude higher than the other sites, with a CER_avg_ of 11.2 × 10^−3^ and a CER_max_ of 17.7 × 10^−3^ (4.97–17.7 × 10^−3^) ppb per ton.

### 3.2 Health Impact Analysis

**Figure 3:**
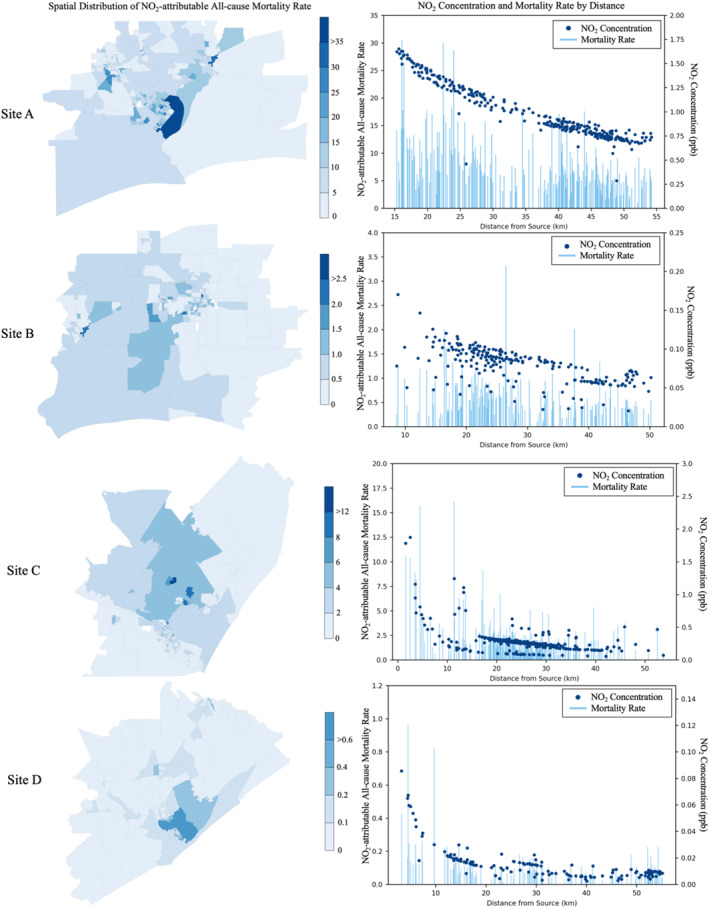
Spatial distribution of block group NO_2_‐attributable all‐cause mortality rate (left panel), NO_2_ concentration and NO_2_‐attributable all‐cause mortality rate by distance of the block‐group centroid from source (right panel). Unit for NO_2_‐attributable all‐cause mortality rate is death per 100,000 people.

We investigated the spatial distribution of NO_2_‐attributable mortality rates (Figure 3, left panel) and compared the health impacts to modeled NO_2_ concentrations by distance from the LNG facility (Figure 3, right panel; and Figure S4 in Supporting Information S1). The overall distribution of mortality rates across block groups exhibited a clustering pattern similar to the concentration distribution in Figure 2. Sites A and B both observed additional clusters further from the source with high mortality rates. At Site A, block groups with the highest mortality rate (above 30 deaths per 100,000 people) were located approximately 15 km from the source. The highest modeled NO_2_ concentration also occurred at the nearest block group, located 15 km away. At Site B, however, the highest mortality rate was observed at a block group 27 km from the LNG terminal, whereas the highest NO_2_ concentration occurred in block group whose centroid is 9 km from source. This spatial discrepancy may suggest that age distribution and baseline mortality rate could play a larger role affecting the NO_2_‐attributable mortality rate at Site B. Sites C and D followed a similar pattern as Site A, where the highest NO_2_‐attributable mortality rate occurred near the source, within 4 km from each LNG site, respectively. The highest NO_2_ concentration also occurred at the nearest block group approximately within 3 km from the source, respectively.

The variability in NO_2_‐attributable mortality across distance largely reflects underlying demographic differences—particularly age distribution and baseline mortality rates—across block groups. A similar pattern is observed for pediatric asthma: Figure S4 in Supporting Information S1 shows that NO_2_‐attributable pediatric asthma rates also peak in block groups nearest to each site, within the same distance bands where modeled NO_2_ concentrations are highest. This indicates that the exposure‐driven spatial gradient seen for mortality applies similarly to pediatric respiratory outcomes.

**Figure 4:**
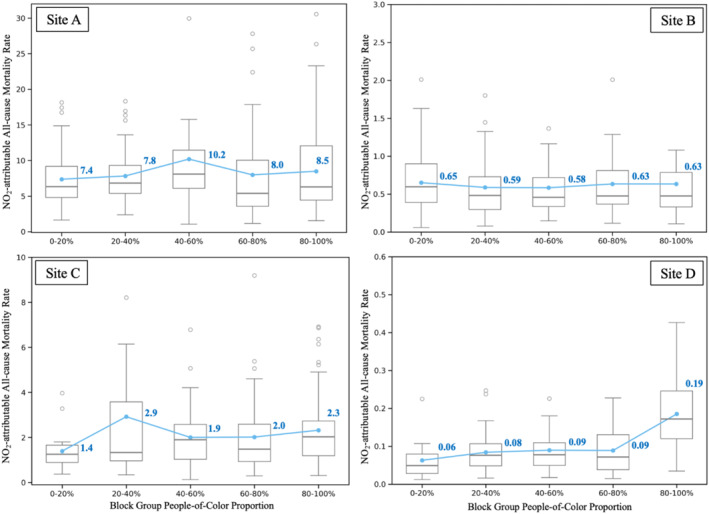
Block group NO_2_‐attributable all‐cause mortality rate (deaths per 100,000 people) by stratified POC% range for Site A (upper left panel), Site B (upper right panel), Site C (lower left panel) and Site D (lower right panel). Blue lines and numbers represent the averages of block group level NO_2_‐attributable mortality rate in each stratified block group POC% range, gray lines represent median values. Upper and lower bounds of the box plots represent 25% and 75% quantiles, respectively. Outliers are marked as circles.

To explore how NO_2_‐attributable mortality rates vary across racial demographics, we stratified block groups into five ranges based on the POC% and compared them, shown in Figure 4. The NO_2_‐attributable all‐cause mortality rate is in annual deaths per 100,000 people living within 50 km to the LNG sites. Site‐level differences emerged in both the magnitude and distribution of mortality rates across POC% ranges. At Site A, the highest mortality rate occurred in block groups with 40%–60% POC%, averaging 10.2 deaths per 100,000 people, while the lowest rate was found in the 0%–20% range (7.4 deaths per 100,000). This pattern suggests that communities with moderate‐to‐high POC% may face a disproportionate burden of NO_2_‐attributable mortality. At Site B, the highest mortality rate occurred in block groups with 0%–20% POC%, averaging 0.65 deaths per 100,000 people, while the lowest rate was in the 40%–60% group (0.58 deaths per 100,000). This higher mortality rate in the low‐POC% range also aligned with the predominantly White demographic pattern reflected by Figure 2. At Site C, the highest mortality rate occurred in the 20%–40% POC% range, averaging 2.9 deaths per 100,000 people, while the lowest rate was in the 0%–20% POC% range (1.4 deaths per 100,000). At Site D, the highest mortality rate occurred in the 80%–100% POC% range, averaging 0.2 deaths per 100,000 people, while the lowest rate was in the 40%–60% range, averaging 0.06 deaths per 100,000. These stratified mortality estimates, particularly Sites A, C, and D, all show an overall increasing trend, aligning with the exposure disparities observed in later equity analysis, as well as in Figure S5 in Supporting Information S1, where stratified exposures by POC% groups show the same upward trends.

### 3.3 Equity Analysis

Figure 2 (middle and bottom panels) shows the spatial distribution of POC% and LI% across block groups surrounding each LNG site. Unlike the modeled NO_2_ concentration patterns, POC% and LI% do not display consistent gradients near the LNG terminals. At Site A, POC% and LI% show three clustering areas including one located near the facility, while block groups near Site B tend to have lower values. Site C shows higher overall POC% and LI% with limited spatial variability, whereas Site D exhibits a cluster with higher POC% and LI% near the source. Overall, demographic patterns around the LNG facilities reflect broader regional sociodemographic distributions rather than localized clustering around the terminals.

To demonstrate equity implications, Table 1 summarizes NO_2_ concentrations, demographic characteristics, and health burdens across distance quintiles at each site. Overall, NO_2_ concentrations consistently decline with distance, but the demographic and health patterns by distance quintile differ by site. Additionally, age structure shows little variation across distance quintiles at all four sites, with elderly population ranging between 12% and 19% of total population. At Site A, the nearest quintile (Q1) shows the highest modeled NO_2_ (10.8 ppb) and the highest socioeconomic indicators (POC% = 82%, LI% = 47%), as well as the largest health burdens (mortality, 11.7 deaths/100,000; pediatric asthma, 112.8 cases/100,000). By contrast, the farthest quintile (Q5) has the lowest NO_2_ (6.3 ppb) and lower POC% (44%) and LI% (33%), with mortality (5.0) and asthma (52.7) roughly half the nearest‐quintile values. Site D is similar to Site A, where the nearest quintile shows the highest modeled NO_2_ (0.26 ppb), POC% (65%), LI% (42%), mortality (0.2) and asthma (2.3) as compared to the farthest quintile. Sites B and C show more mixed patterns. Site B's closest quintile (Q1) has modest NO_2_ (1.0 ppb) and relatively low POC% (17%) and LI% (25%), while mid‐quintiles show higher POC% (53% in Q3) and LI% (47%) even though NO_2_ declines. Site C has high POC% across quintiles (66% in Q1, 57% in Q5) with NO_2_ falling from 4.9 ppb (Q1) to 2.1 ppb (Q5). Health burdens also decline from mortality 3.7 to 1.6 and asthma 33.0 to 14.8 per 100,000. This suggests potential disproportionate exposures and health burdens for block groups with higher proportions of people of color and low‐income population at Sites A and D, but is not observed at Sites B and C.

**Table 1:** Summary of Modeled NO_2_ Levels, Demographic Characteristics, and Health Burdens by Distance Quintiles for Each Site

Site	Distance quintile	Distance km	NO_2_ concentration ppb	POC (%)	LI (%)	Elderly population (%)	Mortality rate deaths/100,000	Pediatric asthma rate cases/100,000
Site A	Q1	18.8	10.8	82	47	14	11.7	112.8
	Q2	27.3	8.5	28	28	16	9.5	84.2
	Q3	39.3	6.6	60	41	17	7.5	66.1
	Q4	44.9	6.5	54	38	16	6.9	61.8
	Q5	50.0	6.3	44	33	17	5.0	52.7
Site B	Q1	16.3	1.0	17	25	15	0.73	7.5
	Q2	22.0	0.8	43	42	15	0.73	7.7
	Q3	25.9	0.6	53	47	18	0.73	6.1
	Q4	34.1	0.6	35	34	14	0.50	5.0
	Q5	44.5	0.5	23	36	17	0.46	4.6
Site C	Q1	13.9	4.9	66	44	17	3.7	33.0
	Q2	21.6	3.0	77	46	17	2.3	22.0
	Q3	24.5	2.7	73	42	15	1.7	20.5
	Q4	27.8	2.5	62	28	15	1.6	18.6
	Q5	36.7	2.1	57	30	19	1.6	14.8
Site D	Q1	10.5	0.26	65	42	12	0.21	2.3
	Q2	19.9	0.22	42	27	13	0.08	1.0
	Q3	30.4	0.20	37	27	16	0.10	0.9
	Q4	42.4	0.16	40	22	18	0.05	0.5
	Q5	53.0	0.16	39	29	17	0.08	0.6

*Note.* For each site and each distance quintile, the reported values in this table are calculated as the mean of all block groups within the same quintile.

**Figure 5:**
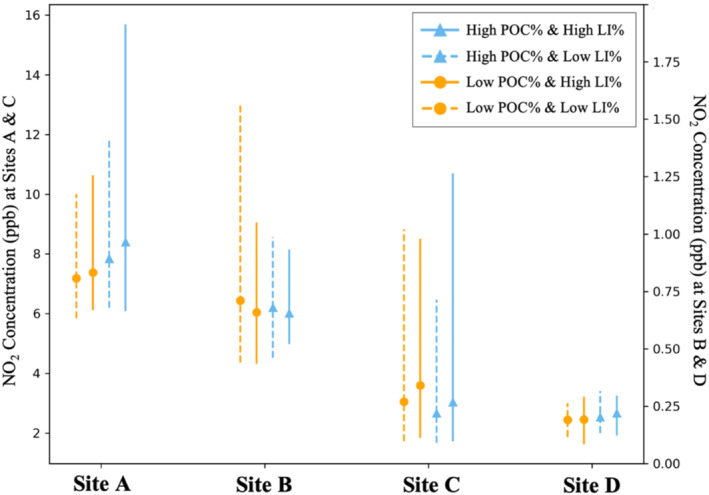
Range and mean values of modeled NO_2_ concentrations by block‐group level intersectional racial‐socioeconomic groups, categorized as the High POC% and High LI% (solid blue line with triangle), High POC% and Low LI% (dashed blue line with triangle), Low POC% and High LI% (solid orange line with circle), and Low POC% and Low LI% (dashed orange line with circle). Dual *y*‐axis is customized to fit the magnitude of modeled concentrations.

To further assess the equity implications of NO_2_ exposures, we examined NO_2_ concentrations by the intersection of race and income across sites. We stratified block groups according to both the percentage of people of color (POC%) and the percentage of low‐income population (LI%). Block groups were categorized into four groups based on the mean POC% and LI% at each site: high POC%–high LI%, high POC%–low LI%, low POC%–high LI%, and low POC%–low LI%. As shown in Figure 5, the highest modeled NO_2_ concentrations were generally observed in block groups with both high POC% and high LI%, particularly near Sites A and D, with mean NO_2_ concentrations of 8.40 (A) and 0.22 (D) ppb compared to 7.19 (A) and 0.19 (D) ppb among low POC% and low LI% groups. At Site C, block groups with either high POC% or high LI% consistently experienced the higher exposure burdens, with 3.03 and 3.60 ppb compared to 2.67 and 3.05 ppb. This pattern suggests disproportionate exposure burdens at the intersection of racial and socioeconomic status. In contrast, Site B was the only exception: here, the low POC% and low LI% group had the highest mean NO_2_ concentrations (0.71 ppb) than the high POC% and high LI% group (0.66 ppb), underscoring that local context can alter the direction of disparities. This pattern also aligns with the negative correlations revealed in Table 1. Overall, these findings indicate that exposure inequities are not limited to a single demographic factor, but can be amplified at the racial–socioeconomic intersection, where non‐white and low‐income groups experience disproportionately higher NO_2_ exposure. Site B is the only exception here, highlighting the importance of site‐specific demographic context.

## 4 Discussion

This study applies the EPA AERMOD model to evaluate near‐source equity and health impacts of NO_*x*_‐emitting facilities at the block group level. We first simulated NO_2_ dispersion from four LNG export terminals using the AERMOD model. We then conducted equity analysis by performing linear regressions between NO_2_ exposures and the proportion of people of color and low‐income population in each block group, to examine potential exposure disparities. Finally, we evaluated the health impacts by quantifying NO_2_‐attributable all‐cause mortality within each block group.

Among the four LNG sites, Site A resulted in the highest daily 1‐hr maximum NO_2_ concentration, primarily due to its highest NO_*x*_ emissions. None of the modeled concentrations exceeded the EPA NAAQS: the highest daily 1‐hr maximum NO_2_ concentration (15.7 ppb at Site A) represents approximately 16% of the NAAQS (100 ppb for the 1‐hr daily maximum). The ratio of maximum to minimum modeled concentrations was consistent across sites, ranging from a factor of three (Sites A and D) to six (Site C). Our results also showed alignments between emission magnitude and concentration levels across most sites (Sites A‐C), measured as Concentration‐to‐Emission Ratios (CER). CER_avg_ values ranged from 1.35 × 10^−3^ to 11.2 × 10^−3^ ppb per ton, and CER_max_ ranged from 3.11 × 10^−3^ to 17.7 × 10^−3^. These results demonstrate consistent proportional relationships between maximum and minimum concentrations, as well as between emissions and modeled concentrations.

To estimate the health risks as granularly as possible, we used census‐tract level age‐specific baseline mortality rates combined with block‐group‐level age‐stratified population data. Health impacts, measured as NO_2_‐attributable all‐cause mortality were substantial, totaling 34.4 deaths (95% CI: 17.4–51.0) across all sites, with block groups near Site A burdened about 70% of the total. Estimated NO_2_‐attributable mortality rates ranged from 1 to 43 deaths per 100,000 people at Site A (average: 8.2), 0 to 3 at Site B (average: 0.6), 0 to 16 at Site C (average: 2.2), and 0 to 1 at Site D (average: 0.1). We also estimated NO_2_‐attributable pediatric asthma cases (for age group 0–14 years) as another health endpoint, totaling 75.9 cases (95% CI: 31.5–102.9) across four sites. Estimated NO_2_‐attributable pediatric asthma rates due to LNG emissions ranged from 0 to 203 cases per 100,000 children under 14 years old at Site A (average: 75.5), 0 to 15 at Site B (average: 6.2), 0 to 152 at Site C (average: 21.8), and 0 to 5 at Site D (average: 1.1).

We further examined NO_2_‐attributable mortality rates by stratifying block groups by POC% ranges. The site‐specific mortality rates by POC% range revealed health disparity pattern at Sites A, C, and D, where mortality rates showed an overall increasing trend with POC%. In contrast, Site B showed a slightly opposite trend, aligning with the results of equity analyses.

Our results also show that populations closest to LNG export terminals do not necessarily result in higher NO_2_‐attributable health impacts. At all four sites, the highest NO_2_ concentrations occurred at the nearest block groups (3–15 km from the LNG facilities, depending on centroid location). However, elevated mortality rates and pediatric asthma rates were observed both near and far from the LNG sites. While elevated mortality and pediatric asthma risks near the source are primarily driven by higher NO_2_ concentrations, high mortality and pediatric asthma rates in more distant block groups occur when age distribution or baseline incidence rates play a larger role. In the case of NO_2_‐attributable all‐cause mortality, further stratification showed that the proportion of elderly population—a demographic with substantially higher baseline mortality rates—varied the most than distance from source and NO_2_ exposure between the higher‐ and lower‐burdened groups, contributing to differences in estimated mortality. As a result, even with lower concentrations and greater distances, some block groups with older populations experienced disproportionately high NO_2_‐attributable mortality.

To contextualize our findings, we compare our results with the study led by Camilleri et al. On a per capita basis, our mortality estimates fall within the range of NO_2_‐attributable all‐cause mortality quantified by prior research. Camilleri et al. estimated that NO_2_ concentrations, resulted from all‐sourced NO_*x*_ emissions, contribute to 170,850 premature deaths annually across the contiguous U.S., equivalent to 90 deaths per 100,000 people per year (Camilleri, Montgomery, et al., 2023). Our results estimated approximately 2.8 deaths per 100,000 people annually, based only on LNG emissions.

Comparing with the LNG‐specific health impact analysis by Greenpeace and the Sierra Club, our estimated total annual mortality of 34.4 deaths attributed to LNG NO_*x*_ emissions is about 60% of the 60 annual premature deaths from regional pollution, as estimated for all operating LNG terminals in 2024 in their analysis (Heureaux‐Torres et al., 2024). Such differences can be explained by several factors. First, the studies differ fundamentally in health outcome endpoints. While our analysis quantified NO_2_‐attributable all‐cause mortality from direct NO_2_ exposure, the previous study estimated premature deaths associated with secondary PM_2.5_ and ozone exposures. Second, methodological differences, including spatial resolution, exposure assessment models, health impact functions, baseline mortality rates, and population demographics, further contribute to variations in mortality estimates. Specifically, our study used the AERMOD model to assess near‐source pollutant concentrations and health impacts at the block‐group level within a 50 km radius of LNG export terminals. The Greenpeace and Sierra Club study employed the EPA's COBRA model, which estimates health impacts at the county and state levels. As noted by the authors, COBRA assumes uniform pollutant exposure across a county, which may lead to underestimation by averaging out peak concentrations in highly burdened areas. Overall, our modeling methodology and health impact analysis yield results that highlight the importance of spatially resolved pollution and health data in generating more granular estimates of near‐source health outcomes. Given the differences in scope and scale, the approach we took serves to complement previous studies.

We conducted regression analyses to investigate potential disparities in NO_2_ exposure. The distance‐quintile analysis indicates that equity patterns vary across sites due to differences in emissions, local demographics, facility siting, and wind‐driven dispersion. At Sites A and D, the nearest communities exhibit the highest NO_2_ concentrations along with higher proportions of People of Color and low‐income population and greater modeled health burdens, suggesting localized spatial inequities. In contrast, Sites B and C show mixed demographic patterns that do not align consistently with distance or NO_2_ levels, likely reflecting the local demographic pattern of predominantly white populations or high‐income near these facilities. To supplement this assessment, we also performed OLS, SLM, and SEM tests (Table S3 in Supporting Information S1). While OLS results show negligible demographic associations at most sites (Sites B, C, D), strong spatial autocorrelation indicates that simple linear regressions are unreliable. After accounting for spatial dependence using SLM and SEM, demographic coefficients generally diminish, suggesting that NO_2_ variation is largely driven by spatial processes—such as emissions magnitude, terrain, and meteorology—rather than demographic gradients. Overall, these findings indicate localized inequities at Sites A and D, but no strong or consistent demographic patterns across all sites.

We acknowledge certain limitations in the modeling approach, especially the treatment of the plume interacting with, and adding to, the broader chemical environment of the region (U.S. EPA, 2024b, 2024d). LNG emissions react with chemicals in the local and regional environment to form ground‐level ozone and secondary PM_2.5_, adding to the health risks of LNG export terminals to nearby communities. We did not model these secondary pollutants, as AERMOD is not designed to capture secondary pollutant formation associated with the plume interacting with background constituents. Our analysis focuses on NO_2_ from the four LNG export terminals and does not account for contributions from other local or regional sources of NO_*x*_. Background NO_2_ from traffic, industrial facilities, or other nearby sources would be expected to influence ambient concentrations and total health burden. Our results should be interpreted as the incremental impact of the LNG export terminals above typical background levels. Additionally, this analysis focuses exclusively on LNG export terminals, rather than all types of LNG‐related facilities, to evaluate representative operational conditions and community impacts.

AERMOD's performance can be influenced by the representativeness of meteorological fields generated by AERMET. As a steady‐state Gaussian plume model, it generally performs better for longer averaging periods than for short‐term concentrations, and uncertainties can arise near sources or under complex atmospheric conditions (Qian & Venkatram, 2011; Rood, 2014). In this study, we did not directly evaluate model predictions against monitoring data as this was out of the scope. Instead, we used standard AERMET configurations and surface and upper‐air observations recommended by U.S. EPA. We note that the modeled concentrations should be interpreted as estimates of overall magnitude and spatial patterns, and that subsequent exposure and health analyses may carry uncertainty driven by meteorological representativeness and boundary layer characterization.

In our health impact analyses, we quantified NO_2_‐attributable all‐cause mortality and pediatric asthma as health endpoints due to their well‐documented health impacts, but other NO_2_ health impacts increased risks of include cardiovascular diseases, cancer, reproductive and developmental effects (Anenberg et al., 2022; U.S. EPA, 2016), as well as a wide range of impacts from emitted pollutants beyond NO_2_, and chemically formed ozone and PM_2.5_. It should also be noted that pediatric asthma is typically estimated for individuals under age 18, but in this study, we used the under‐14 age group due to the available population data age breakdown. Additionally, while the census‐tract‐level baseline mortality rate and state‐level baseline pediatric asthma incidence rate represent the finest‐resolution data available, they may not fully capture variability across block groups. Furthermore, our health impact estimates for long‐term NO_2_ exposure rely on CRFs derived primarily from studies of traffic‐related air pollution. While the HEI CRF (RR = 1.04 per 10 μg/m^3^) is broadly consistent with pooled estimates in the literature (Faustini et al., 2014), it is important to recognize that NO_2_ often serves as a marker for a complex mixture of traffic‐related pollutants, including ultrafine particles and other co‐pollutants. Emissions from LNG terminals differ in composition and source characteristics from urban traffic, meaning that applying this CRF introduces uncertainty regarding the specific causal effects of NO_2_ from these facilities.

Finally, while our analysis estimated block‐group‐level NO_2_ exposure, it is important to note that finer spatial resolution does not always translate to improved accuracy of individual exposure estimates. People move throughout the day for work, school, or other activities, so exposure at the home location may not fully represent total exposure (Nyhan et al., 2016). Additionally, proportional allocation of population within census units assumes uniform distribution, which may not reflect true population patterns in areas with heterogeneous land use (Michanowicz et al., 2019). These factors might introduce uncertainty in the exposure estimates, highlighting the distinction between precision (high spatial resolution) and accuracy (closeness to true personal exposure) in environmental justice assessments.

## 5 Conclusion

This study develops and applies a novel, computationally‐efficient modeling framework to evaluate near‐source pollution dispersion, equity impacts, and health risks. The methodology developed here highlights the capability of regulatory models such as AERMOD to evaluate fine‐scale pollution dispersion near high‐emitting facilities like LNG export terminals. This approach provides a novel way to examine potential disparities in pollution exposure and health risks burdened by disadvantaged communities, offering critical insights into environmental justice implications. The NO_2_ emissions examined here are not unique to LNG sources; rather, they result from fuel combustion more broadly, including vehicles, buildings, industrial heat, and electricity generation. Similar mortality concerns apply to regions where elevated NO_2_ concentrations overlap with densely populated areas, particularly affecting elderly and other vulnerable populations.

## Conflict of Interest

The authors declare no conflicts of interest relevant to this study.

## Supporting information

Supporting Information S1

## Data Availability

All data used in this study are publicly available, and the sources are cited in the manuscript. Meteorological data were obtained from the National Oceanic and Atmospheric Administration (NOAA, 2020a, 2020b). Land cover data and elevation data were obtained from the U.S. Geological Survey (USGS, 2020a, 2020b). Emissions data were obtained from the U.S. Environmental Protection Agency (EPA, 2025a). Demographic data were obtained from the U.S. Census Bureau (2020). Baseline mortality data were obtained from the EPA Environmental Benefits Mapping and Analysis Program USALEEP database (U.S. EPA, 2025e), and baseline pediatric asthma data were obtained from the Global Burden of Disease 2021 study (GBD 2021, 2025). Air quality modeling was conducted using the AERMOD modeling system (U.S. EPA, 2024e). The scripts used for analysis and figure generation is available via the GitHub repository *LNG_AERMOD_Health* and has been archived on Zenodo (X. Wu, 2025).

## References

[gh270119-bib-0001] Abdul‐Wahab, S. , Fadlallah, S. , Al‐Riyami, M. , Alsouti, M. S. , & Osman, I. (2020). A study of the effects of CO, NO_2_, and PM10 emissions from the Oman Liquefied Natural Gas (LNG) plant on ambient air quality. Air Quality, Atmosphere & Health, 13(6), 1235–1245. 10.1007/s11869-020-00876-w

[gh270119-bib-0002] Achakulwisut, P. , Brauer, M. , Hystad, P. , & Anenberg, S. C. (2019). Global, national, and urban burdens of paediatric asthma incidence attributable to ambient NO_2_ pollution: Estimates from global datasets. The Lancet Planetary Health, 3(4), e166–e178. 10.1016/S2542-5196(19)30046-4 30981709

[gh270119-bib-0003] Adeniran, J. A. , Yusuf, R. O. , Fakinle, B. S. , & Sonibare, J. A. (2019). Air quality assessment and modelling of pollutants emission from a major cement plant complex in Nigeria. Atmospheric Pollution Research, 10(1), 257–266. 10.1016/j.apr.2018.07.010

[gh270119-bib-0004] Al‐Yafei, H. , Kucukvar, M. , AlNouss, A. , Aseel, S. , & Onat, N. C. (2021). A novel hybrid life cycle assessment approach to air emissions and human health impacts of liquefied natural gas supply chain. Energies, 14(19), 19. 10.3390/en14196278

[gh270119-bib-0005] Amoatey, P. , Omidvarborna, H. , Baawain, M. S. , & Al‐Mamun, A. (2020). Evaluation of vehicular pollution levels using line source model for hot spots in Muscat, Oman. Environmental Science and Pollution Research, 27(25), 31184–31201. 10.1007/s11356-020-09215-z 32488708

[gh270119-bib-0006] Anenberg, S. C. , Mohegh, A. , Goldberg, D. L. , Kerr, G. H. , Brauer, M. , Burkart, K. , et al. (2022). Long‐term trends in urban NO2 concentrations and associated paediatric asthma incidence: Estimates from global datasets. The Lancet Planetary Health, 6(1), e49–e58. 10.1016/S2542-5196(21)00255-2 34998460

[gh270119-bib-0007] Arani, M. H. , Jaafarzadeh, N. , Moslemzadeh, M. , Rezvani Ghalhari, M. , Bagheri Arani, S. , & Mohammadzadeh, M. (2021). Dispersion of NO_2_ and SO_2_ pollutants in the rolling industry with AERMOD model: A case study to assess human health risk. Journal of Environmental Health Science and Engineering, 19(2), 1287–1298. 10.1007/s40201-021-00686-x 34900266 PMC8617121

[gh270119-bib-0008] Baker, E. , Goldstein, A. P. , & Azevedo, I. M. (2021). A perspective on equity implications of net zero energy systems. Energy and Climate Change, 2, 100047. 10.1016/j.egycc.2021.100047

[gh270119-bib-0009] Banzhaf, S. , Ma, L. , & Timmins, C. (2019). Environmental justice: The economics of race, place, and pollution. The Journal of Economic Perspectives, 33(1), 185–208. 10.1257/jep.33.1.185 30707005

[gh270119-bib-0010] Bazilian, M. D. , Carley, S. , Konisky, D. , Zerriffi, H. , Pai, S. , & Handler, B. (2021). Expanding the scope of just transitions: Towards localized solutions and community‐level dynamics. Energy Research & Social Science, 80, 102245. 10.1016/j.erss.2021.102245

[gh270119-bib-0011] Bell, M. L. , & Ebisu, K. (2012). Environmental inequality in exposures to airborne particulate matter components in the United States. Environmental Health Perspectives, 120(12), 1699–1704. 10.1289/ehp.1205201 22889745 PMC3546368

[gh270119-bib-0012] Boningari, T. , & Smirniotis, P. G. (2016). Impact of nitrogen oxides on the environment and human health: Mn‐based materials for the NO_x_ abatement. Current Opinion in Chemical Engineering, 13, 133–141. 10.1016/j.coche.2016.09.004

[gh270119-bib-0013] Bullard, R. D. (2018). Dumping in dixie: Race, class, and environmental quality, third edition (3rd ed.). Routledge. 10.4324/9780429495274

[gh270119-bib-0014] Buonocore, J. J. , Reka, S. , Yang, D. , Chang, C. , Roy, A. , Thompson, T. , et al. (2023). Air pollution and health impacts of oil & gas production in the United States. Environmental Research: Health, 1(2), 021006. 10.1088/2752-5309/acc886

[gh270119-bib-0015] Camilleri, S. F. , Kerr, G. H. , Anenberg, S. C. , & Horton, D. E. (2023). All‐Cause NO2‐Attributable mortality burden and associated racial and ethnic disparities in the United States. Environmental Science and Technology Letters, 10(12), 1159–1164. 10.1021/acs.estlett.3c00500 38106529 PMC10720462

[gh270119-bib-0016] Camilleri, S. F. , Montgomery, A. , Visa, M. A. , Schnell, J. L. , Adelman, Z. E. , Janssen, M. , et al. (2023). Air quality, health and equity implications of electrifying heavy‐duty vehicles. Nature Sustainability, 6(12), 1643–1653. 10.1038/s41893-023-01219-0

[gh270119-bib-0017] Carley, S. , & Konisky, D. M. (2020). The justice and equity implications of the clean energy transition. Nature Energy, 5(8), 569–577. 10.1038/s41560-020-0641-6

[gh270119-bib-0018] Chen, X. , Qi, L. , Li, S. , & Duan, X. (2024). Long‐term NO2 exposure and mortality: A comprehensive meta‐analysis. Environmental Pollution, 341, 122971. 10.1016/j.envpol.2023.122971 37984474

[gh270119-bib-0019] Cimorelli, A. J. , Perry, S. G. , Venkatram, A. , Weil, J. C. , Paine, R. J. , Wilson, R. B. , et al. (2005). AERMOD: A dispersion model for industrial source applications. Part I: General model formulation and boundary layer characterization. 10.1175/JAM2227.1

[gh270119-bib-0020] Clark, L. P. , Harris, M. H. , Apte, J. S. , & Marshall, J. D. (2022). National and Intraurban Air pollution exposure disparity estimates in the United States: Impact of data‐aggregation spatial Scale. Environmental Science and Technology Letters, 9(9), 786–791. 10.1021/acs.estlett.2c00403 36118958 PMC9476666

[gh270119-bib-0021] Collett, J. L. , Pan, D. , McKenzie, L. , Zimmerle, D. , Zhang, W. , Zhou, Y. , et al. (2025). Measuring and modeling air pollution and noise exposure near unconventional oil and gas development in Colorado (research report no. 232). HEI. Retrieved from https://www.healtheffects.org/system/files/collett‐research‐report‐232‐report.pdf PMC13036433 41913658

[gh270119-bib-0022] Craig, K. J. , Baringer, L. M. , Chang, S.‐Y. , McCarthy, M. C. , Bai, S. , Seagram, A. F. , et al. (2020). Modeled and measured near‐road PM2.5 concentrations: Indianapolis and Providence cases. Atmospheric Environment, 240, 117775. 10.1016/j.atmosenv.2020.117775

[gh270119-bib-0023] Cushing, L. J. , Li, S. , Steiger, B. B. , & Casey, J. A. (2023). Historical red‐lining is associated with fossil fuel power plant siting and present‐day inequalities in air pollutant emissions. Nature Energy, 8(1), 52–61. 10.1038/s41560-022-01162-y 41797883 PMC12965463

[gh270119-bib-0024] Donaghy, T. Q. , Healy, N. , Jiang, C. Y. , & Battle, C. P. (2023). Fossil fuel racism in the United States: How phasing out coal, oil, and gas can protect communities. Energy Research & Social Science, 100, 103104. 10.1016/j.erss.2023.103104

[gh270119-bib-0025] Doost, Z. E. , Dehghani, S. , Samaei, M. R. , Arabzadeh, M. , Baghapour, M. A. , Hashemi, H. , et al. (2024). Dispersion of SO_2_ emissions in a gas refinery by AERMOD modeling and human health risk: A case study in the Middle East. International Journal of Environmental Health Research, 34(2), 1227–1240. 10.1080/09603123.2023.2165044 36682061

[gh270119-bib-0026] Epstein, P. R. , Buonocore, J. J. , Eckerle, K. , Hendryx, M. , Stout III, B. M. , Heinberg, R. , et al. (2011). Full cost accounting for the life cycle of coal. Annals of the New York Academy of Sciences, 1219(1), 73–98. 10.1111/j.1749-6632.2010.05890.x 21332493

[gh270119-bib-0027] Faustini, A. , Rapp, R. , & Forastiere, F. (2014). Nitrogen dioxide and mortality: Review and meta‐analysis of long‐term studies. European Respiratory Journal, 44(3), 744–753. 10.1183/09031936.00114713 24558178

[gh270119-bib-0028] Galvin, R. (2020). “Let justice roll down like waters”: Reconnecting energy justice to its roots in the civil rights movement. Energy Research & Social Science, 62, 101385. 10.1016/j.erss.2019.101385

[gh270119-bib-0029] Gardner‐Frolick, R. , Boyd, D. , & Giang, A. (2022). Selecting data analytic and modeling methods to support air pollution and environmental justice investigations: A critical review and guidance framework. Environmental Science & Technology, 56(5), 2843–2860. 10.1021/acs.est.1c01739 35133145

[gh270119-bib-0030] GBD 2021 . (2025). Institute for Health Metrics and evaluation (IHME). Retrieved from http://vizhub.healthdata.org/gbd‐compare

[gh270119-bib-0031] Gohlke, J. M. , Harris, M. H. , Roy, A. , Thompson, T. M. , DePaola, M. , Alvarez, R. A. , et al. (2023). State‐of‐the‐science data and methods need to guide place‐based efforts to reduce air pollution inequity. Environmental Health Perspectives, 131(12), 125003. 10.1289/EHP13063 38109120 PMC10727036

[gh270119-bib-0032] Gutiérrez, C. G. , Martínez, Á. H. , de Navamuel, E. D. R. , Piris, A. O. , & Rojo, B. B. (2025). Atmospheric emission assessment for LNG carriers: A state‐of‐the‐art semi‐empirical methodology. Environmental Science and Pollution Research International, 32(9), 5419–5434. 10.1007/s11356-025-36037-8 39930098 PMC11868231

[gh270119-bib-0033] Health Effects Institute . (2022). Systematic review and meta‐analysis of selected health effects of long‐term exposure to traffic‐related air pollution (no. HEI special report 23). Retrieved from https://www.healtheffects.org/system/files/hei‐special‐report‐23_6.pdf

[gh270119-bib-0034] Heureaux‐Torres, J. , Chang, A. , & Donaghy, T. (2024). Permit to kill: Potential health and economic impacts from U.S. LNG export terminal permitted emissions. Greenpeace and Sierra Club. https://www.greenpeace.org/static/planet4‐usa‐stateless/2024/11/47b90812‐permit‐to‐kill.pdf

[gh270119-bib-0035] Hurbain, P. , Strickland, M. J. , Liu, Y. , & Li, D. (2024). Environmental inequality in estimated cancer risk from airborne toxic exposure across United States communities from 2011 to 2019. Environmental Science & Technology, 58(43), 19115–19127. 10.1021/acs.est.4c02526 39415479 PMC11526371

[gh270119-bib-0036] Jackson, C. M. , Holloway, T. , & Tessum, C. W. (2023). City‐scale analysis of annual ambient PM2.5 source contributions with the InMAP reduced‐complexity air quality model: A case study of Madison, Wisconsin. Environmental Research: Infrastructure and Sustainability, 3(1), 015002. 10.1088/2634-4505/acb0fa

[gh270119-bib-0037] Johnson, G. S. , Washington, S. C. , King, D. W. , & Gomez, J. M. (2014). Air quality and health issues along Houston’s ship channel: An exploratory environmental justice analysis of a vulnerable community (Pleasantville). Race, Gender & Class, 21(3/4), 273–303.

[gh270119-bib-0038] Johnston, J. E. , Lim, E. , & Roh, H. (2019). Impact of upstream oil extraction and environmental public health: A review of the evidence. Science of the Total Environment, 657, 187–199. 10.1016/j.scitotenv.2018.11.483 30537580 PMC6344296

[gh270119-bib-0039] Josimović, B. , Todorović, D. , Jovović, A. , & Manić, B. (2024). Air pollution modeling to support strategic environmental assessment: Case study—National Emission Reduction Plan for coal‐fired thermal power plants in Serbia. Environment, Development and Sustainability, 26(6), 16249–16265. 10.1007/s10668-023-03186-0

[gh270119-bib-0040] Jung, C.‐R. , Lin, Y.‐T. , & Hwang, B.‐F. (2015). Ozone, particulate matter, and newly diagnosed alzheimer’s disease: A population‐based cohort study in Taiwan. Journal of Alzheimer's Disease, 44(2), 573–584. 10.3233/JAD-140855 25310992

[gh270119-bib-0041] Kemfert, C. , Präger, F. , Braunger, I. , Hoffart, F. M. , & Brauers, H. (2022). The expansion of natural gas infrastructure puts energy transitions at risk. Nature Energy, 7(7), 582–587. 10.1038/s41560-022-01060-3

[gh270119-bib-0042] Kerr, G. H. , Goldberg, D. L. , & Anenberg, S. C. (2021). COVID‐19 pandemic reveals persistent disparities in nitrogen dioxide pollution. Proceedings of the National Academy of Sciences, 118(30), e2022409118. 10.1073/pnas.2022409118 PMC8325165 34285070

[gh270119-bib-0043] Kirrane, E. F. , Bowman, C. , Davis, J. A. , Hoppin, J. A. , Blair, A. , Chen, H. , et al. (2015). Associations of ozone and PM2.5 concentrations with Parkinson’s disease among participants in the agricultural health study. Journal of Occupational and Environmental Medicine, 57(5), 509–517. 10.1097/JOM.0000000000000451 25951420 PMC4428683

[gh270119-bib-0044] Kumar, A. , Patil, R. S. , Dikshit, A. K. , & Kumar, R. (2017). Application of AERMOD for short‐term air quality prediction with forecasted meteorology using WRF model. Clean Technologies and Environmental Policy, 19(7), 1955–1965. 10.1007/s10098-017-1379-0

[gh270119-bib-0045] Lewis, B. M. , Battye, W. H. , Aneja, V. P. , Kim, H. , & Bell, M. L. (2023). Modeling and analysis of air pollution and environmental justice: The case for North Carolina’s Hog concentrated animal feeding operations. Environmental Health Perspectives, 131(8), 087018. 10.1289/EHP11344 37616159 PMC10449010

[gh270119-bib-0046] Liu, J. , Clark, L. P. , Bechle, M. J. , Hajat, A. , Kim, S.‐Y. , Robinson, A. L. , et al. (2021). Disparities in air pollution exposure in the United States by race/ethnicity and income, 1990–2010. Environmental Health Perspectives, 129(12), 127005. 10.1289/EHP8584 34908495 PMC8672803

[gh270119-bib-0047] Liu, T. , Chan, A. W. H. , & Abbatt, J. P. D. (2021). Multiphase oxidation of sulfur dioxide in aerosol particles: Implications for sulfate formation in polluted environments. Environmental Science & Technology, 55(8), 4227–4242. 10.1021/acs.est.0c06496 33760581

[gh270119-bib-0048] Maroko, A. R. (2012). Using air dispersion modeling and proximity analysis to assess chronic exposure to fine particulate matter and environmental justice in New York city. Applied Geography, 34, 533–547. 10.1016/j.apgeog.2012.02.005

[gh270119-bib-0049] Mathieu, M. E. , Gray, J. , & Richmond‐Bryant, J. (2023). Spatial associations of long‐term exposure to diesel particulate matter with seasonal and annual mortality due to COVID‐19 in the contiguous United States. BMC Public Health, 23(1), 423. 10.1186/s12889-023-15064-5 36869295 PMC9982169

[gh270119-bib-0050] Mauzerall, D. L. , Sultan, B. , Kim, N. , & Bradford, D. F. (2005). NO_x_ emissions from large point sources: Variability in ozone production, resulting health damages and economic costs. Atmospheric Environment, 39(16), 2851–2866. 10.1016/j.atmosenv.2004.12.041

[gh270119-bib-0051] Michanowicz, D. R. , Williams, S. R. , Buonocore, J. J. , Rowland, S. T. , Konschnik, K. E. , Goho, S. A. , & Bernstein, A. S. (2019). Population allocation at the housing unit level: Estimates around underground natural gas storage wells in PA, OH, NY, WV, MI, and CA. Environmental Health, 18(1), 58. 10.1186/s12940-019-0497-z 31280723 PMC6613251

[gh270119-bib-0052] Mikati, I. , Benson, A. F. , Luben, T. J. , Sacks, J. D. , & Richmond‐Bryant, J. (2018). Disparities in distribution of particulate matter emission sources by race and poverty status. American Journal of Public Health, 108(4), 480–485. 10.2105/AJPH.2017.304297 29470121 PMC5844406

[gh270119-bib-0053] Mohai, P. , Pellow, D. , & Roberts, J. T. (2009). Environmental justice. Annual Review of Environment and Resources, 34(1), 405–430. 10.1146/annurev-environ-082508-094348

[gh270119-bib-0054] Murphy, C. F. , & Allen, D. T. (2005). Hydrocarbon emissions from industrial release events in the Houston‐Galveston area and their impact on ozone formation. Atmospheric Environment, 39(21), 3785–3798. 10.1016/j.atmosenv.2005.02.051

[gh270119-bib-0055] National Center for Health Statistics . (2025). U.S. small‐area life expectancy estimates project—USALEEP. Retrieved from https://www.cdc.gov/nchs/nvss/usaleep/usaleep.html

[gh270119-bib-0056] NOAA . (2020a). Hourly surface observation data. Retrieved from https://www1.ncdc.noaa.gov/pub/data/noaa/

[gh270119-bib-0057] NOAA . (2020b). Upper air radiosonde data. Retrieved from https://ruc.noaa.gov/raobs/

[gh270119-bib-0058] Nyhan, M. , Grauwin, S. , Britter, R. , Misstear, B. , McNabola, A. , Laden, F. , et al. (2016). “Exposure Track”—The impact of mobile‐device‐based mobility patterns on quantifying population exposure to air pollution. Environmental Science & Technology, 50(17), 9671–9681. 10.1021/acs.est.6b02385 27518311

[gh270119-bib-0059] Oh, J. , Kim, S. , Kim, M. S. , Abate, Y. H. , ElHafeez, S. A. , Abdelkader, A. , et al. (2025). Global, regional, and national burden of asthma and atopic dermatitis, 1990–2021, and projections to 2050: A systematic analysis of the global Burden of Disease Study 2021. The Lancet Respiratory Medicine, 13(5), 425–446. 10.1016/S2213-2600(25)00003-7 40147466

[gh270119-bib-0060] O’Rourke, D. , & Connolly, S. (2003). Just oil? The distribution of environmental and social impacts of oil production and consumption. Annual Review of Environment and Resources, 28(2), 587–617. 10.1146/annurev.energy.28.050302.105617

[gh270119-bib-0061] Pandey, G. , Venkatram, A. , & Arunachalam, S. (2023). Evaluating AERMOD with measurements from a major U.S. airport located on a shoreline. Atmospheric Environment, 294, 119506. 10.1016/j.atmosenv.2022.119506

[gh270119-bib-0062] Paolella, D. A. , Tessum, C. W. , Adams, P. J. , Apte, J. S. , Chambliss, S. , Hill, J. , et al. (2018). Effect of model spatial resolution on estimates of fine particulate matter exposure and exposure disparities in the United States. Environmental Science and Technology Letters, 5(7), 436–441. 10.1021/acs.estlett.8b00279

[gh270119-bib-0063] Peel, J. L. , Haeuber, R. , Garcia, V. , Russell, A. G. , & Neas, L. (2013). Impact of nitrogen and climate change interactions on ambient air pollution and human health. Biogeochemistry, 114(1), 121–134. 10.1007/s10533-012-9782-4

[gh270119-bib-0064] Perry, S. G. , Cimorelli, A. J. , Paine, R. J. , Brode, R. W. , Weil, J. C. , Venkatram, A. , et al. (2005). AERMOD: A dispersion model for industrial source applications. Part II: Model performance against 17 field Study databases. 10.1175/JAM2228.1

[gh270119-bib-0065] Pospíšil, J. , Charvát, P. , Arsenyeva, O. , Klimeš, L. , Špiláček, M. , & Klemeš, J. J. (2019). Energy demand of liquefaction and regasification of natural gas and the potential of LNG for operative thermal energy storage. Renewable and Sustainable Energy Reviews, 99, 1–15. 10.1016/j.rser.2018.09.027

[gh270119-bib-0066] Qian, W. , & Venkatram, A. (2011). Performance of steady‐state dispersion models under low wind‐speed conditions. Boundary‐Layer Meteorology, 138(3), 475–491. 10.1007/s10546-010-9565-1

[gh270119-bib-0067] Radford, A. , Geddes, J. A. , Gallagher, K. , & Larson, B. A. (2021). Open‐source methods for estimating health risks of fine particulate matter from coal‐fired power plants: A demonstration from Karachi, Pakistan. Environmental Impact Assessment Review, 91, 106638. 10.1016/j.eiar.2021.106638

[gh270119-bib-0068] Ragothaman, A. , & Anderson, W. A. (2017). Air quality impacts of petroleum refining and petrochemical industries. Environments, 4(3), 3. 10.3390/environments4030066

[gh270119-bib-0069] Rood, A. S. (2014). Performance evaluation of AERMOD, CALPUFF, and legacy air dispersion models using the Winter Validation Tracer Study dataset. Atmospheric Environment, 89, 707–720. 10.1016/j.atmosenv.2014.02.054

[gh270119-bib-0070] Rosselot, K. S. , Balcombe, P. , Ravikumar, A. P. , & Allen, D. T. (2023). Simulating the variability of methane and CO2 emissions from liquefied natural gas shipping: A time‐in‐mode and carrier technology approach. ACS Sustainable Chemistry & Engineering, 11(43), 15632–15643. 10.1021/acssuschemeng.3c04269

[gh270119-bib-0071] Ruszel, M. (2022). The development of global LNG exports. In K. Liuhto (Ed.), The future of energy consumption, security and natural gas: LNG in the Baltic Sea region (pp. 1–20). Springer International Publishing. 10.1007/978-3-030-80367-4_1

[gh270119-bib-0072] Saha, R. , Bullard, R. D. , & Powers, L. T. (2024). Liquefying the Gulf Coast: A cumulative impact assessment of LNG buildout in Louisiana and Texas. Bullard Center for Environmental and Climate Justice. Retrieved from https://www.bullardcenter.org/resources/liquefied‐natural‐gas‐lng

[gh270119-bib-0073] Salva, J. , Vanek, M. , Schwarz, M. , Gajtanska, M. , Tonhauzer, P. , & Ďuricová, A. (2021). An assessment of the on‐road mobile sources contribution to particulate matter air pollution by AERMOD dispersion model. Sustainability, 13(22), 22. 10.3390/su132212748

[gh270119-bib-0074] Scolio, M. , Borha, C. , Kremer, P. , & Shakya, K. M. (2024). Spatial analysis of intra‐urban air pollution disparities through an environmental justice lens: A case Study of Philadelphia, PA. Atmosphere, 15(7), 755. 10.3390/atmos15070755

[gh270119-bib-0075] Shan, X. , Casey, J. A. , Shearston, J. A. , & Henneman, L. R. F. (2024). Methods for quantifying source‐specific air pollution exposure to serve epidemiology, risk assessment, and environmental justice. GeoHealth, 8(11), e2024GH001188. 10.1029/2024GH001188 PMC11536408 39502358

[gh270119-bib-0076] Southerland, V. A. , Anenberg, S. C. , Harris, M. , Apte, J. , Hystad, P. , van Donkelaar, A. , et al. (2021). Assessing the distribution of air pollution health risks within cities: A neighborhood‐scale analysis leveraging high‐resolution data sets in the Bay Area, California. Environmental Health Perspectives, 129(3), 037006. 10.1289/EHP7679 33787320 PMC8011332

[gh270119-bib-0077] Stocker, J. , Seaton, M. , Smith, S. , O’Neill, J. , Johnson, K. , Jackson, R. , & Carruthers, D. (2023). Evaluation of the generic reaction set method for NO_2_ conversion in AERMOD. Cambridge Environmental Research Consultants (CERC). Retrieved from https://gaftp.epa.gov/Air/aqmg/SCRAM/conferences/2023_13th_Conference_On_Air_Quality_Modeling/Review_Material/20230808_GRSM_Evaluation_Report_CERC.pdf

[gh270119-bib-0078] Terrell, K. A. , & Julien, G. S. (2023). Discriminatory outcomes of industrial air permitting in Louisiana, United States. Environmental Challenges, 10, 100672. 10.1016/j.envc.2022.100672

[gh270119-bib-0079] Terrell, K. A. , & St Julien, G. (2022). Air pollution is linked to higher cancer rates among black or impoverished communities in Louisiana. Environmental Research Letters, 17(1), 014033. 10.1088/1748-9326/ac4360

[gh270119-bib-0080] Tessum, C. , Hill, J. , & Marshall, J. (2016). InMAP: A new model for air pollution health impact assessments. ISEE Conference Abstracts, 2016(1), P2–P394. 10.1289/isee.2016.4739

[gh270119-bib-0081] Tessum, C. W. , Hill, J. D. , & Marshall, J. D. (2017). InMAP: A model for air pollution interventions. PLoS One, 12(4), e0176131. 10.1371/journal.pone.0176131 28423049 PMC5397056

[gh270119-bib-0082] Tessum, C. W. , Paolella, D. A. , Chambliss, S. E. , Apte, J. S. , Hill, J. D. , & Marshall, J. D. (2021). PM2.5 polluters disproportionately and systemically affect people of color in the United States. Science Advances, 7(18), eabf4491. 10.1126/sciadv.abf4491 33910895 PMC11426197

[gh270119-bib-0083] Tsai, J.‐H. , Gu, W.‐T. , Chung, I.‐I. , & Chiang, H.‐L. (2019). Airborne air toxics characteristics and inhalation health risk assessment of a metropolitan industrial complex. Aerosol and Air Quality Research, 19(11), 247–2489. 10.4209/aaqr.2019.08.0422

[gh270119-bib-0084] Turner, M. C. , Jerrett, M. , Pope, C. A. , Krewski, D. , Gapstur, S. M. , Diver, W. R. , et al. (2016). Long‐Term ozone exposure and mortality in a large prospective Study. American Journal of Respiratory and Critical Care Medicine, 193(10), 1134–1142. 10.1164/rccm.201508-1633OC 26680605 PMC4872664

[gh270119-bib-0085] U.S. Census Bureau . (2020). American Community Service (ACS) 2020 data. Retrieved from https://data.census.gov/table

[gh270119-bib-0086] U.S. Department of Energy . (2014). Life cycle greenhouse gas perspective on exporting liquefied natural gas from the United States. Retrieved from https://www.energy.gov/fecm/articles/life‐cycle‐greenhouse‐gas‐perspective‐exporting‐liquefied‐natural‐gas‐united‐states

[gh270119-bib-0087] U.S. Department of Energy . (2024). Energy, economic, and environmental assessment of U.S. LNG exports. Retrieved from https://www.energy.gov/sites/default/files/2024‐12/LNGUpdate_SummaryReport_Dec2024_230pm.pdf

[gh270119-bib-0088] U.S. EPA . (2004). AERMOD: Description of model formulation (no. EPA‐454/R‐03‐004). Retrieved from https://gaftp.epa.gov/aqmg/SCRAM/models/preferred/aermod/aermod_mfd_454‐R‐03‐004.pdf

[gh270119-bib-0089] U.S. EPA . (2016). Integrated Science Assessment (ISA) for oxides of nitrogen—Health criteria (final report) (no. EPA/600/R‐15/068). U.S. Environmental Protection Agency. Retrieved from https://cfpub.epa.gov/ncea/isa/recordisplay.cfm?deid=310879

[gh270119-bib-0090] U.S. EPA . (2022). User’s guide for the AERMOD meteorological preprocessor (AERMET) (User guide no. EPA‐454/B‐22‐006). Retrieved from https://gaftp.epa.gov/Air/aqmg/SCRAM/models/met/aermet/aermet_userguide.pdf

[gh270119-bib-0091] U.S. EPA . (2024a). Environmental justice mapping and screening tool EJScreen technical documentation for version 2.3. Retrieved from https://www.epa.gov/system/files/documents/2024‐07/ejscreen‐tech‐doc‐version‐2‐3.pdf

[gh270119-bib-0092] U.S. EPA . (2024b). Guidance on developing background concentrations for use in modeling demonstrations (no. EPA‐454/R‐24‐003). Retrieved from https://www.epa.gov/system/files/documents/2024‐11/background‐concentrations.pdf

[gh270119-bib-0093] U.S. EPA . (2024c). Guideline on air quality models (Appendix W to 40 CFR Part 51). Retrieved from https://www.epa.gov/scram/2024‐appendix‐w‐final‐rule

[gh270119-bib-0094] U.S. EPA . (2024d). Guideline on air quality models; enhancements to the AERMOD dispersion modeling system (rule Nos. 2024‐27636 (89 FR 95034)). Federal Register. Retrieved from https://www.federalregister.gov/documents/2024/11/29/2024‐27636/guideline‐on‐air‐quality‐models‐enhancements‐to‐the‐aermod‐dispersion‐modeling‐system

[gh270119-bib-0095] U.S. EPA . (2025a). 2022v2 emissions modeling platform. Retrieved from https://www.epa.gov/air‐emissions‐modeling/2022v2‐emissions‐modeling‐platform

[gh270119-bib-0096] U.S. EPA . (2025b). Air data: Air quality data collected at outdoor monitors across the US [Dataset]. Retrieved from https://www.epa.gov/outdoor‐air‐quality‐data

[gh270119-bib-0097] U.S. EPA . (2025c). CMAQ: The community multiscale air quality modeling System. Retrieved from https://www.epa.gov/cmaq

[gh270119-bib-0098] U.S. EPA . (2025d). CO‐Benefits risk assessment health impacts screening and mapping tool (COBRA). Retrieved from https://www.epa.gov/cobra

[gh270119-bib-0099] U.S. EPA . (2025e). Environmental benefits mapping and analysis Program—Community edition (BenMAP‐CE). Retrieved from https://www.epa.gov/benmap

[gh270119-bib-0100] U.S. EPA . (2025f). Environmental justice screening and mapping tool (EJScreen).

[gh270119-bib-0101] USGS . (2020a). National elevation data. Retrieved from https://apps.nationalmap.gov/downloader/

[gh270119-bib-0102] USGS . (2020b). National land cover database. Retrieved from https://www.mrlc.gov/viewer/

[gh270119-bib-0103] Valencia, A. , Serre, M. , & Arunachalam, S. (2023). A hyperlocal hybrid data fusion near‐road PM2.5 and NO_2_ annual risk and environmental justice assessment across the United States. PLoS One, 18(6), e0286406. 10.1371/journal.pone.0286406 37262039 PMC10234552

[gh270119-bib-0104] Williams, S. B. , Shan, Y. , Jazzar, U. , Kerr, P. S. , Okereke, I. , Klimberg, V. S. , et al. (2020). Proximity to oil refineries and risk of cancer: A population‐based analysis. JNCI Cancer Spectrum, 4(6), pkaa088. 10.1093/jncics/pkaa088 33269338 PMC7691047

[gh270119-bib-0105] Wu, R. , Tessum, C. W. , Zhang, Y. , Hong, C. , Zheng, Y. , Qin, X. , et al. (2021). Reduced‐complexity air quality intervention modeling over China: The development of InMAPv1.6.1‐China and a comparison with CMAQv5.2. Geoscientific Model Development, 14(12), 7621–7638. 10.5194/gmd-14-7621-2021

[gh270119-bib-0106] Wu, X. (2025). LNG_AERMOD_Health (version 1.0) [Software]. Zenodo. 10.5281/zenodo.17373298

[gh270119-bib-0107] Yuan, T.‐H. , Shen, Y.‐C. , Shie, R.‐H. , Hung, S.‐H. , Chen, C.‐F. , & Chan, C.‐C. (2018). Increased cancers among residents living in the neighborhood of a petrochemical complex: A 12‐year retrospective cohort study. International Journal of Hygiene and Environmental Health, 221(2), 308–314. 10.1016/j.ijheh.2017.12.004 29287935

[gh270119-bib-0108] Zhang, R. , Gen, M. , Liang, Z. , Li, Y. J. , & Chan, C. K. (2022). Photochemical reactions of glyoxal during particulate ammonium nitrate photolysis: Brown carbon Formation, enhanced glyoxal decay, and organic phase Formation. Environmental Science & Technology, 56(3), 1605–1614. 10.1021/acs.est.1c07211 35023733

[gh270119-bib-0109] Zou, Q. , Yi, C. , Wang, K. , Yin, X. , & Zhang, Y. (2022). Global LNG market: Supply‐demand and economic analysis. IOP Conference Series: Earth and Environmental Science, 983(1), 012051. 10.1088/1755-1315/983/1/012051

