# Supplementary material for: Assessing Near‐Source Health and Equity Impacts of Liquefied Natural Gas Terminals
Source: Geohealth. 2026 Apr 13;10(4):e2025GH001609. doi: 10.1029/2025GH001609 (PMC13072054; doi:10.1029/2025GH001609)
Supplement: Supplementary file 1 — Supporting Information S1 [file GH2-10-e2025GH001609-s001.pdf]

Supporting Information for

Assessing Near-Source Health and Equity Impacts of Liquefied Natural Gas Terminals

Xinran Wu[1*], Tracey Holloway[1,2], Drashti Amin[1], Paul Meier[1], Vijay Limaye[3,4], Ade Samuel[5]

[1] Nelson Institute Center for Sustainability and the Global Environment, University of Wisconsin-Madison, Madison, WI, USA
[2] Department of Atmospheric and Oceanic Sciences, University of Wisconsin-Madison, Madison, WI, USA
[3] Natural Resources Defense Council, New York, NY, USA
[4] Department of Population Health Sciences, University of Wisconsin-Madison, Madison, WI, USA
[5] Natural Resources Defense Council, Washington, D.C., USA

Contents of this file

Tables S1 to S3

Figures S1 to S5

Text S1 to S2

Table S1: Summary of site-specific emissions, demographic, stack characteristics, meteorological stations by LNG site

Site	2022 EMP NO_X Emissions tons / year	Block groups included	Total Population	Stack Parameters				Background Ozone ppb	Upper Air	Surface NWS
				Height	Temp.	Velocity	Diameter			
**Site A**	4266.6	279	324,940	49.4	684	20.5	2.9	36.8	03937	03937
**Site B**	505.1	208	253,808	43.2	736	31.3	3.6	36.7	03937	03937
**Site C**	2045.3	338	446,489	64	690	26.9	3.0	33.6	12924	12924
**Site D**	17.9	137	194,628	7.4	752	44.9	1.1	37.8	12960	12960

Stack characteristics were used as inputs to AERMOD, data from 2022 EPA Emission Modeling Platform: stack height in m, stack exit temperature in K, stack exit velocity in m/s, and stack diameter in m.

Table S2: Summary of modeled results by LNG site

Site	NO_2 Concentration			Total Mortality			Mortality Rate			Total Pediatric Asthma			Pediatric Asthma Rate		
	Annual Mean	Min	Max	Mean	Lower 95% CI	Upper 95% CI	Mean	Min	Max	Mean	Lower 95% CI	Upper 95% CI	Mean	Min	Max
**Site A**	0.99	5.8	15.7	23.5	11.9	34.9	8.2	1.1	42.5	51.3	21.3	69.4	75.5	0.0	203.4
**Site B**	0.08	0.4	1.6	1.5	0.8	2.2	0.6	0.1	3.3	3.3	1.4	4.5	6.2	0.0	14.5
**Site C**	0.29	1.7	10.7	9.2	4.6	13.6	2.2	0.1	16.2	20.9	8.6	28.4	21.8	0.0	152.3
**Site D**	0.02	0.1	0.3	0.2	0.1	0.3	0.1	0	1.0	0.4	0.2	0.6	1.1	0.0	5.4

Concentrations are in ppb at block group level; units for total mortality and pediatric asthma are deaths/cases per year in all block groups modeled within a radius of 50 km; units for mortality and asthma rates are deaths/cases per 100,000 people (children under 14 years) per year at individual block group level.

Table S3: Summary of OLS, SLM, and SEM regression results for equity analysis

Site	Demographic Factors	OLS			Moran's I		SLM			SEM		
		coef	p-value	R^2	I value	p-value	β (coef)	ρ (rho)	p-value	β (coef)	λ (lambda)	p-value
Site A	POC%	1.79	0.000	0.10	0.90	0.001	0.175	0.989	0.000	0.05	0.992	0.000
	LI%	1.85	0.000	0.04	0.90	0.001	0.220	0.991	0.000	-0.004	0.992	0.000
Site B	POC%	-0.08	0.041	0.02	0.79	0.001	-0.016	0.954	0.000	-0.020	0.956	0.000
	LI%	-0.10	0.107	0.01	0.79	0.001	-0.001	0.956	0.000	0.013	0.956	0.000
Site C	POC%	-0.19	0.506	0.00	0.812	0.001	0.019	0.992	0.000	0.048	0.992	0.000
	LI%	0.40	0.213	0.01	0.814	0.001	-0.013	0.992	0.000	0.098	0.992	0.000
Site D	POC%	0.07	0.000	0.13	0.691	0.001	0.002	0.934	0.000	-0.009	0.940	0.000
	LI%	0.05	0.022	0.04	0.773	0.001	-0.006	0.940	0.000	-0.014	0.941	0.000

Text S1: As shown in Table S3, Ordinary Least Squares (OLS) models generally show small to moderate associations between demographic indicators and NO_2 concentrations, with R^2 values ranging from 0.00 to 0.13 across sites. Sites A and D exhibit the strongest OLS relationships, particularly with POC%, while Sites B and C show weak or statistically insignificant coefficients. Importantly, Moran's I statistics for all sites are high ($I \approx 0.69$–0.90) and statistically significant ($p = 0.001$), indicating strong positive spatial autocorrelation in residuals and suggesting that OLS assumptions are not met. These diagnostics justify the use of spatial regression models.

Consistent with the Moran's Is, the Spatial Lag Model (SLM) and Spatial Error Model (SEM) reveal substantial spatial dependence across all sites, with spatial autoregressive parameters (ρ for SLM and λ for SEM) close to 1.0 and statistically significant ($p < 0.001$). In most cases, the magnitude and direction of the demographic coefficients change after accounting for spatial structure, indicating that non-spatial OLS estimates were partially confounded by spatial clustering. At Sites A and D, the SLM and SEM still show positive associations between POC% and LI% and higher modeled NO_2 concentrations. In contrast, Sites B and C show negligible or unstable demographic coefficients once spatial dependence is accounted for, suggesting that spatial processes dominate variation in pollutant levels at those locations.

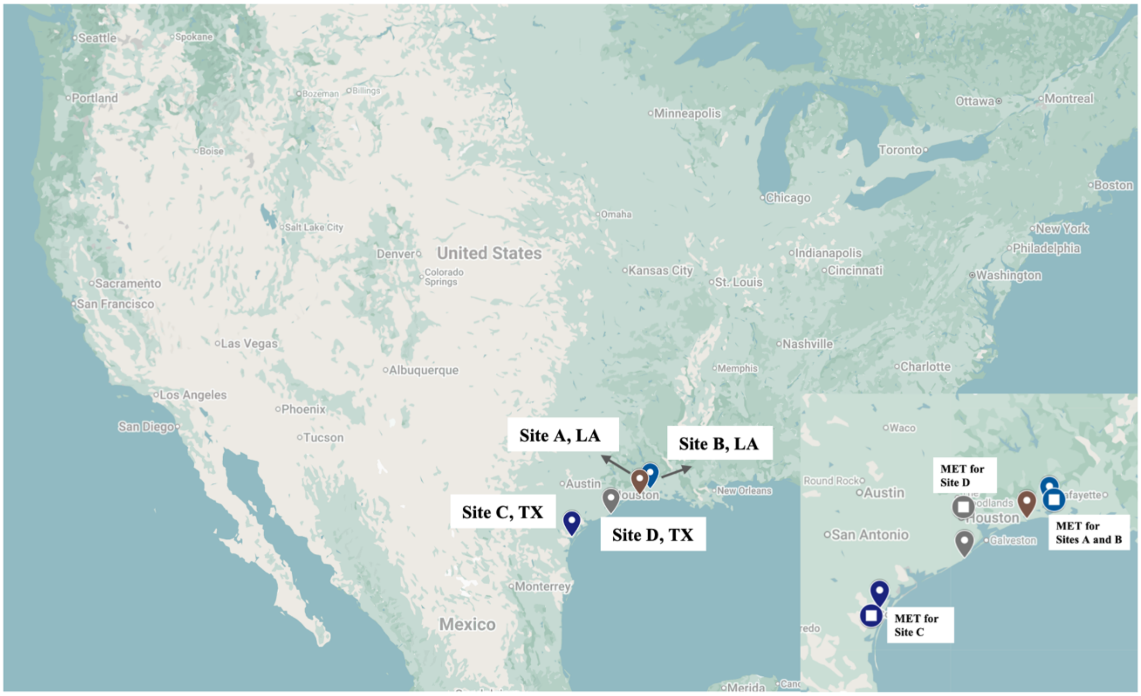

Figure S1. Map of approximate locations of the four LNG export facilities modeled (pinned). Enlarged to show meteorological stations (circled) used for AERMET in this study (bottom right). Basemap adapted from Google Map.

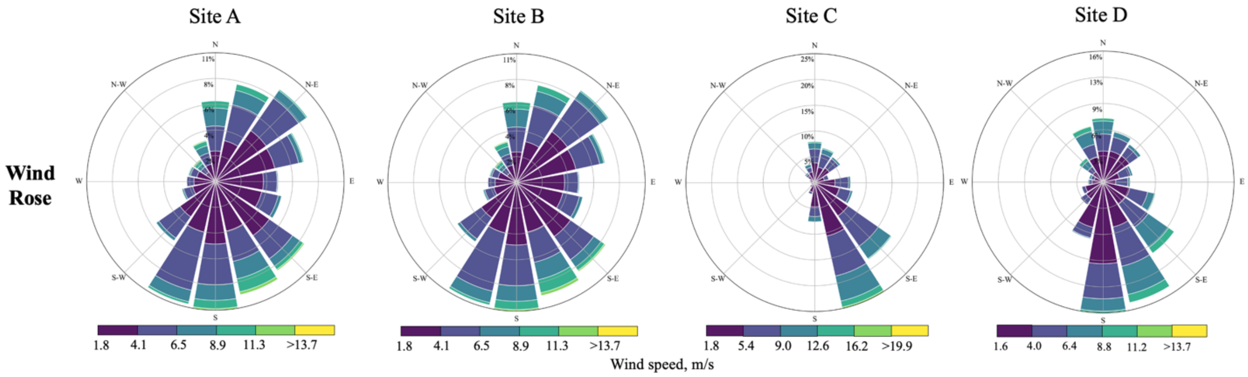

Figure S2. Wind rose diagrams for sites A-D at block group level. Each column represents one LNG site. Each color in the wind rose diagrams represent wind speed in meters per second (m/s), with each radius indicating the frequency (%) of winds from a given direction.

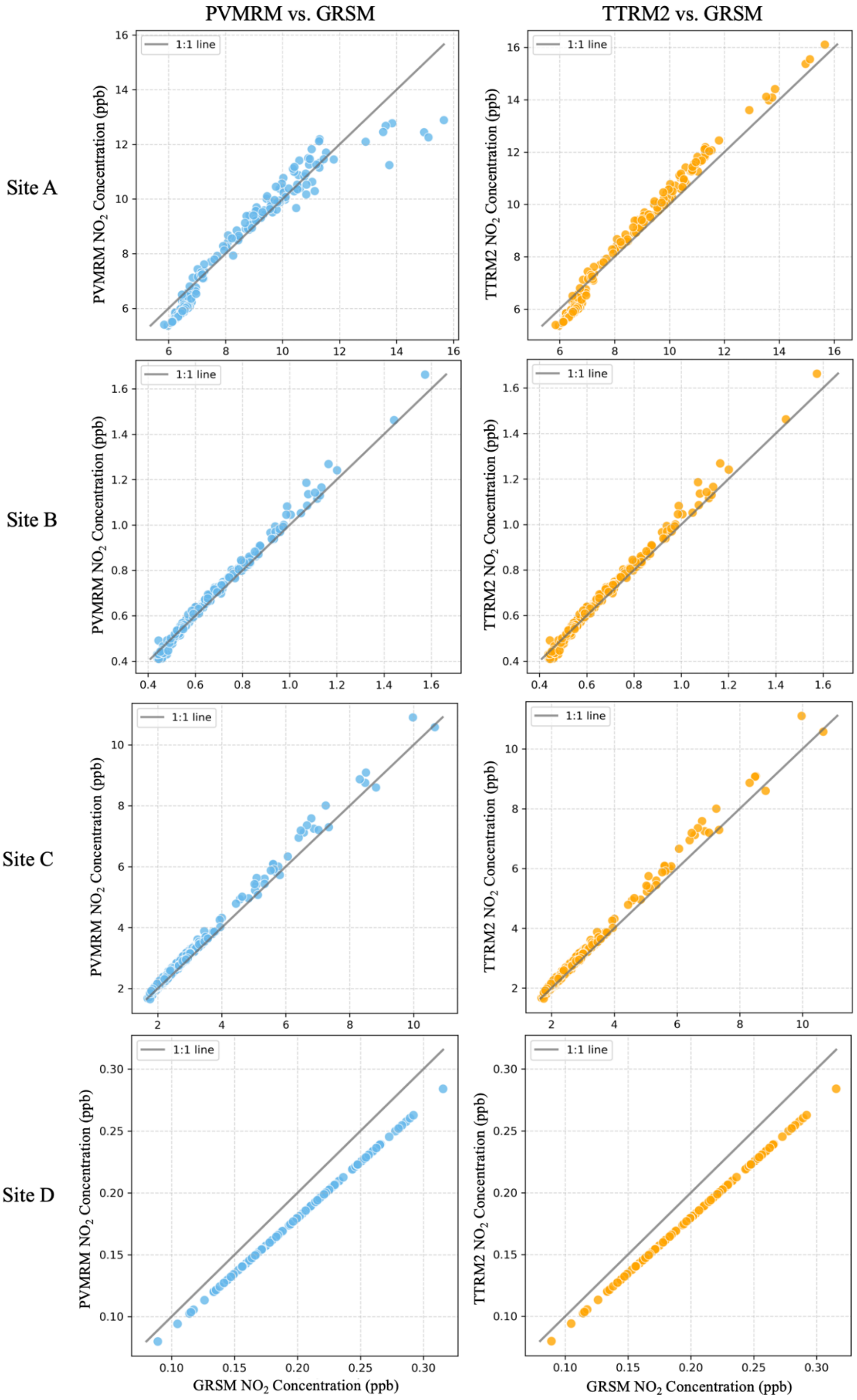

Figure S3. 1:1 line plots comparing modeled 1-hr daily maximum NO_2 concentrations of GRSM-PVMRM schemes (left, blue) and GRSM-TTRM schemes (left, orange), in ppb.

Text S2: Figure S3 shows 1:1 comparison of GRSM with PVMRM (blue) and with TTRM2 (orange) across the four sites. At Site A, daily maximum 1-hr NO_2 concentrations range from 5.8-15.7 ppb (mean, 7.7) for GRSM scheme, 5.4-12.9 ppb (mean, 7.4) for PVMRM scheme, and 5.4-16.1 ppb (mean, 7.6) for TTRM2 scheme; at Site B, 0.4-1.6 ppb (mean, 0.7) for GRSM scheme, 0.4-1.7 ppb (mean, 0.7) for PVMRM scheme, and 0.4-1.7 ppb (mean, 0.7) for TTRM2 scheme; at Site C, 1.7-10.7 ppb (mean, 3.0) for GRSM scheme, 1.7-10.9 ppb (mean, 3.2) for PVMRM scheme, and 1.7-11.1 ppb (mean, 3.2) for TTRM2 scheme; and at Site D, 0.09-0.32 ppb (mean, 0.20) for GRSM scheme, 0.08-0.28 ppb (mean, 0.18) for PVMRM scheme, and 0.08-0.28 ppb (mean, 0.18) for TTRM2 scheme.

In all runs, both PVMRM and TTRM2 schemes tend to overpredict NO_2 concentrations than GRSM, with more scatter points lying above the 1:1 line, except for Site D. Differences between the PVMRM and TTRM2 are negligible especially for low NO2 concentrations modeled at Sites B and D. This pattern indicates that GRSM results fall between the two other schemes and provides a balanced representation for subsequent health and equity analyses.

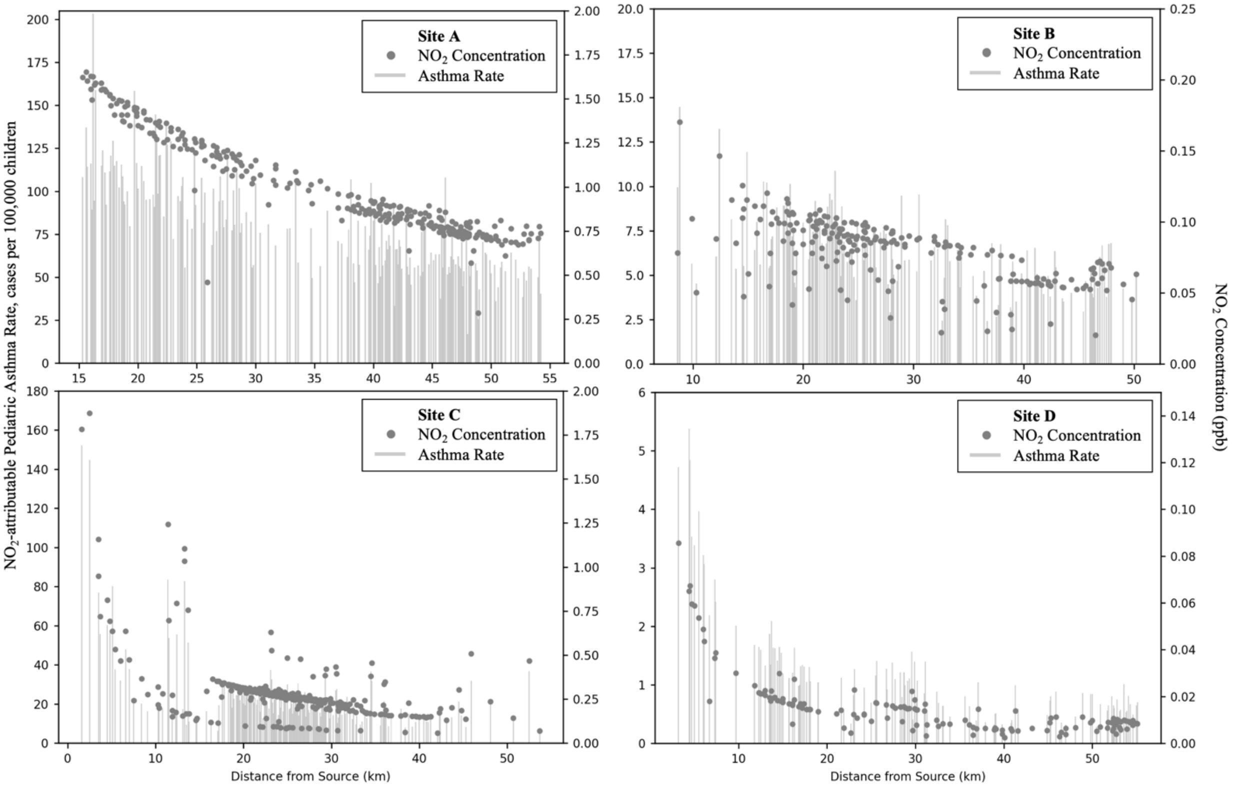

Figure S4. NO_2 concentration and NO_2-attributable pediatric asthma rates by distance of the block-group centroid from source at Site A (upper left panel), Site B (upper right panel), Site C (lower left panel) and Site D (lower right panel). Unit for NO_2-attributable pediatric asthma rates is cases per 100,000 children (under 14 years).

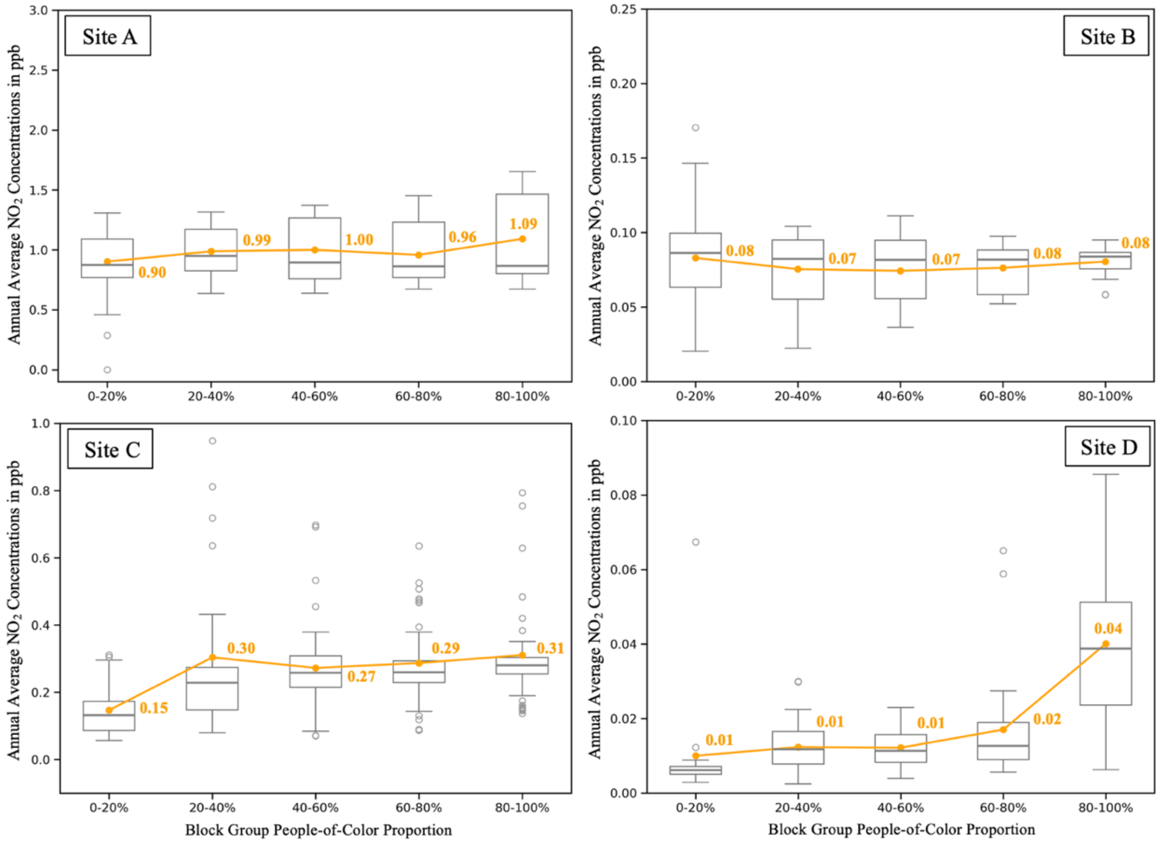

Figure S5. Block-group annual averaged NO_2 concentrations (ppb) by stratified POC% range for Site A (upper left panel), Site B (upper right panel), Site C (lower left panel) and Site D (lower right panel). Orange lines and numbers represent the averages of NO_2 concentrations in each stratified block group POC% range, grey lines represent median values. Upper and lower bounds of the box plots represent 25% and 75% quantiles, respectively. Outliers are marked as circles.
